# Spatial Molecular Signatures of Canine Oral Squamous Cell Carcinoma with and Without Locoregional Metastasis with Cross-Species Comparison to Human Samples

**DOI:** 10.3390/ijms27209163

**Published:** 2026-10-15

**Authors:** Stephanie Goldschmidt, Clifford G. Tepper, Maria Soltero-Rivera, Rachel V. Brady, John Aleman, Robert Rebhun, Xiao-Jing Wang, Andrew C. Birkeland, Karleen Meiklejohn, Ryan Todebusch, Clinson Lui, Brian G. Murphy, Natalia Vapniarsky

**Affiliations:** 1 Department of Surgical & Radiological Sciences, School of Veterinary Medicine, University of California-Davis, Davis, CA 95616, USA; 2 Department of Biochemistry and Molecular Medicine, School of Medicine, University of California-Davis, Davis, CA 95817, USA; 3 Department of Pathology and Laboratory Medicine, School of Medicine, University of California-Davis, Davis, CA 95817, USA; 4 VA Northern California Health Care System, Sacramento, CA 95652, USA; 5 Department of Otolaryngology-Head and Neck Surgery, School of Medicine, University of California-Davis, Davis, CA 95817, USA; 6 Department of Pathology, Microbiology and Immunology, School of Veterinary Medicine, University of California-Davis, Davis, CA 95616, USA

**Keywords:** oral squamous cell carcinoma, head and neck squamous cell carcinoma, locoregional metastasis, transcriptomics, biomarkers

## Abstract

Accurate diagnosis of locoregional spread in head and neck squamous cell carcinoma (HNSCC) is critical for prognosis and treatment planning. However, the currently available pre-surgical staging modalities often fail to detect occult nodal metastasis, necessitating nodal extirpation for accurate pathologic staging. This approach carries no clinical benefit and introduces unwarranted morbidity in patients with nonmetastatic lymph nodes (pN0 neck). The aim of this study was to define the molecular landscape of HNSCC in a spontaneous model of disease (pet dogs) with and without pathologically confirmed locoregional metastasis to identify biomarkers that may improve risk stratification and guide nodal management. Spatial transcriptomic profiling revealed a distinct metastatic tumor signature characterized by downregulation of *PTEN*, *EPSTI1*, *CD99*, *MAPKAPK2*, *OAZ1*, and upregulation of *LOC477699*. The tumor microenvironment of metastatic tumors demonstrated reduced *JAK1* and trends consistent with T-cell exhaustion. Metastatic lymph node deposits conserved transcriptomic signatures matched to the primary tumor, and peri-metastatic lymph nodes displayed potentially permissive molecular alterations, including reduced F13A1 and DLA88 expression. Cross-species comparisons with human datasets identified conserved molecular and pathway-level features. Collectively, these findings identify candidate biomarkers and biological pathways associated with locoregional metastasis and support future prospective validation in canine and human HNSCC.

## 1. Introduction

Accurate diagnosis of locoregional spread for head and neck squamous cell carcinoma (HNSCC) directly impacts prognosis [1,2] and therefore the recommended care paradigm. Yet, current clinically available diagnostic tools including palpation, diagnostic imaging, and fine-needle aspiration continue to underdiagnose occult metastasis due to limited sensitivity or sampling errors [3,4,5,6,7]. Pathology is the gold standard for lymph node staging, with promotion of elective neck dissection and/or sentinel lymph node biopsy being made for accurate prognostication [8,9,10]. Nevertheless, the adoption of pathologic lymph node staging has been slow and continues be controversial regarding its true clinical indications [11,12,13].

In humans, decision analysis has revealed that pathologic staging (lymph node extirpation) of the clinical N0 neck (no evidence of metastasis on non-invasive staging) is indicated when there is a metastatic risk >20% based on the pathologic features of the primary tumor [14]. The strongest pathologic predictor of nodal metastasis is depth of invasion, with >4–5 mm being the optimal cut-off point depending on the study [15,16,17] Other features that are commonly explored with variable predictive strength include the presence of lymphovascular invasion, peripheral nerve invasion, degree of differentiation, and pattern of invasion [18,19,20,21]. Extensive work has also focused on understanding the molecular underpinnings of local regional metastasis with the aim of identifying biomarkers to better guide decision making on nodal dissection as well as identifying targeted therapeutics [22,23,24].

In dogs, pathologic features including tumor-associated inflammation, lymphovascular invasion, perineural invasion [1], proliferating cell nuclear antigen and grade [25] have been correlated with patient outcomes (risk of death and disease-free interval). But neither historical study specifically evaluated pathologic features with the presence of locoregional metastasis, with the latter study having the presence of locoregional metastasis as an exclusion criterion. Further, available genomic and transcriptomic studies in dogs have either included no dogs with lymphatic metastasis or have not clarified if the patients had lymphatic spread as the cohort was pulled from archival samples with incomplete datasets [26,27,28,29]. Collectively, in dogs, there are no identified primary tumor features that risk stratify patients and help guide decision making for nodal dissection in head and neck SCC.

The aim of this study was to define the molecular landscape of HNSCC in dogs with and without pathologically confirmed locoregional metastasis. The sub-aims were to define the molecular landscape of the metastatic niche within the lymph node compared to the primary tumor and normal lymph nodes and compare our results to historical human transcriptomic datasets.

## 2. Results

The overall goal of this study was to define the transcriptomic signatures and associated functional implications of HNSCC in patients with and without locoregional metastasis (pN+ vs. pN0). Digital spatial profiling (DSP) using the GeoMx DSP platform was conducted on primary tumor and nodal samples stratified by metastatic status (pN0 vs. PN+) in 18 dogs (14 with matched tumor and nodal samples for analysis) including comparisons of tumor tissues, TME, and normal peritumoral surface epithelium. Metastatic lymph nodes were compared to the primary tumor and normal lymph nodes. Findings were validated with qualitative RT-PCR. Finally, the canine results were directly compared to human GeoMx samples and TCGA repositories to define cross-species similarities and underscore the translational potential of our pilot findings.

### 2.1. The Primary Tumor’s Aggressive Signature: Distinct Molecular Signatures Are Present in Canine HNSCC with Locoregional Metastasis (pN+ vs. pN0)

**Figure 1:**
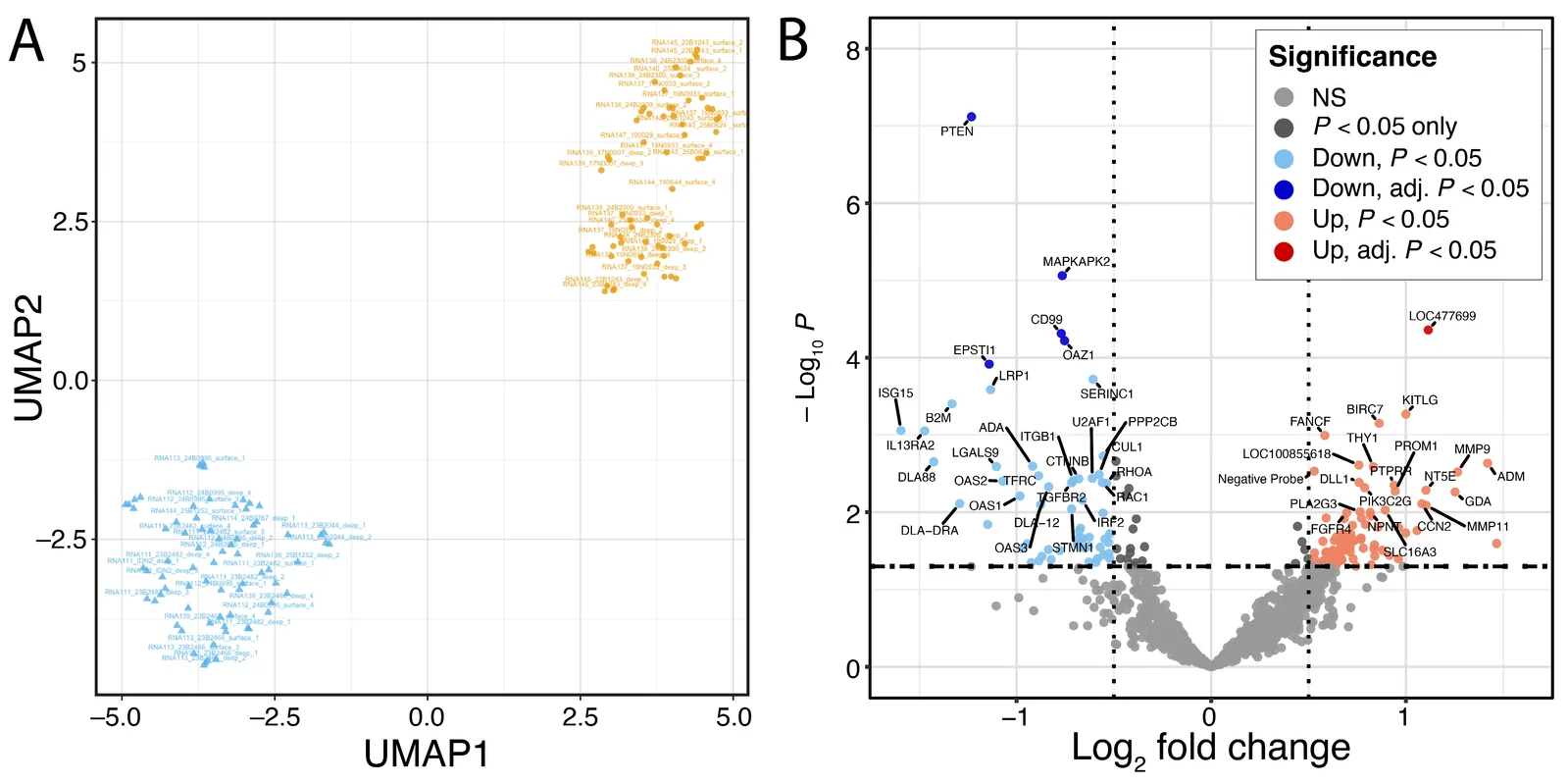
Differentially expressed genes between canine tumors with and without locoregional metastasis (pN0 vs. pN+). (**A**) Distinct clustering of ROI transcript data from metastatic tumor tissue (blue) and nonmetastatic (yellow) tumor tissues is seen on the UMAP visualization. (**B**) Volcano plots depict the upregulated and downregulated differentially expressed genes from group comparisons between metastatic and nonmetastatic tumors. The log2 (fold change) and −log10 (nominal *p* value) are indicated on the x- and y-axes, respectively.

Exploratory visualization of the RNA expression profiles using uniform manifold approximation and projection (UMAP) demonstrated the clustering of primary tumor ROIs that were associated with metastatic status, consistent with differences in gene expression patterns between metastatic and nonmetastatic tumors (Figure 1A). While there was concern that the observed expression differences between pN+ and pN0 could be driven by a DSP-run-associated batch effect, the integrity of the biological findings was verified by a demonstration that potential batch effects were minimal in the dataset both before and after batch correction (Supplementary Figures S1–S5).

Differential expression analysis (limma) identified 150 genes showing nominal differential expression (DEGs, log_2_fold change > 0.5, nominal *p* < 0.05) with 92 being upregulated and 58 downregulated in metastatic HNSCC tumor tissue, respectively (Figure 1B). Following false discovery rate (FDR) correction, 6 genes remained significant (adj. *p* < 0.05) with *PTEN*, *EPSTI1*, *CD99*, *MAPKAPK2*, and *OAZ1* being downregulated and *LOC477699* being upregulated in metastatic tumor tissue.

**Figure 2:**
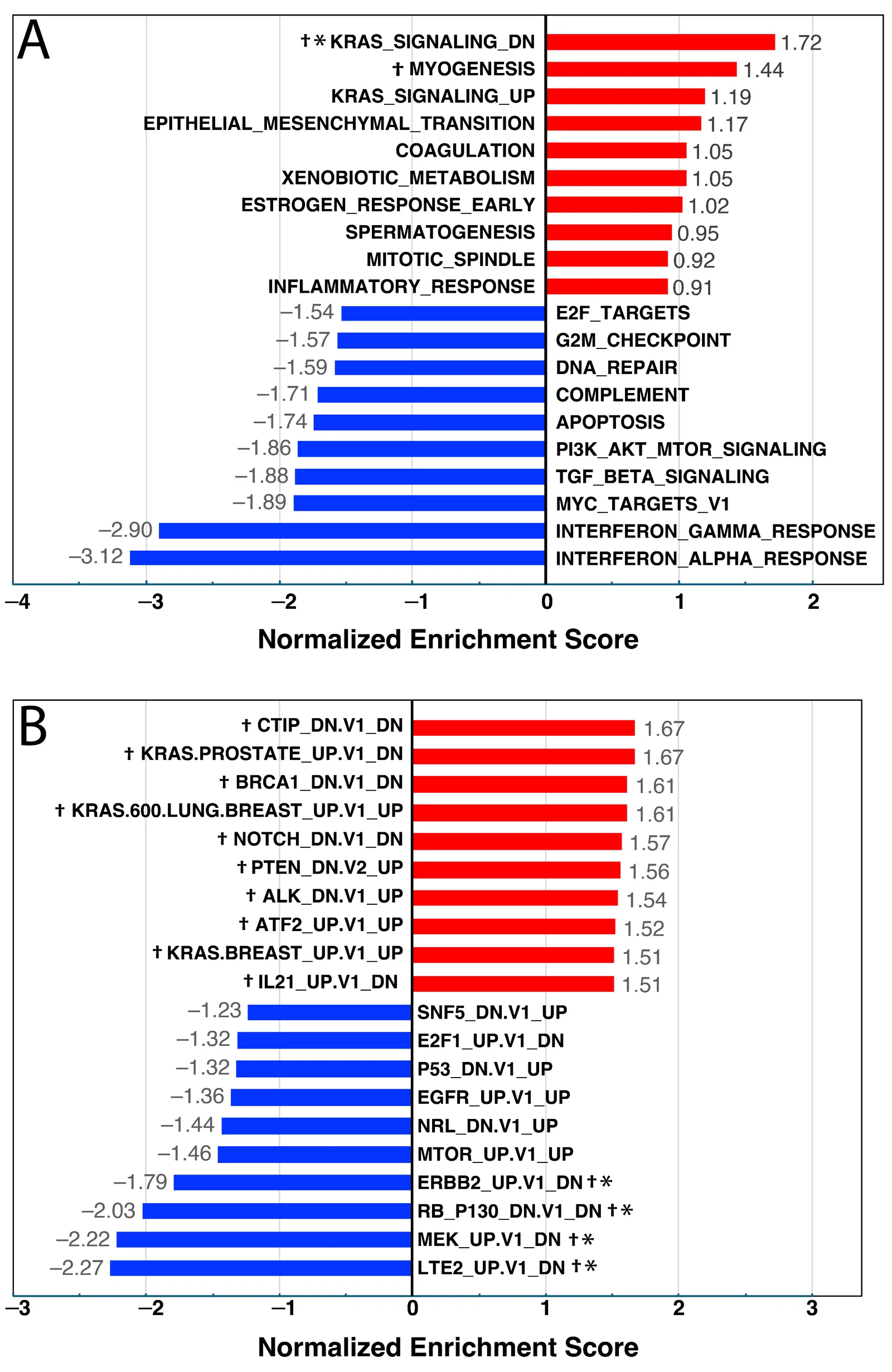
Functional enrichment analyses between canine tumors with and without locoregional metastasis (pN+ vs. pN0). GSEA was performed using the MSigDB Hallmark (**A**) and C6 oncogenic (**B**) gene signature collections. The top 10 upregulated (red) and downregulated (blue) gene sets based on NES are shown. Note: † gene sets significantly enriched with a nominal *p* value < 0.05% * gene sets which were significant at FDR < 25%.

Gene Set Enrichment Analysis (GSEA) was performed to gain insight into the functional implications of the transcriptional alterations between metastatic and nonmetastatic tumors (Figure 2). The most robust enrichments were reductions in interferon alpha and gamma response signatures in metastatic tumors (NES −3.12 and −2.90, q < 0.001). Gene sets associated with PTEN loss, downregulation of the KRAS and PI3K-AKT-MTOR pathways, and activation of MEK pathways showed nominal enrichment in metastatic tumors (nominal *p* < 0.05), and all but PTEN loss retained significance after FDR correction (q < 0.25).

### 2.2. The Permissive Microenvironment and Immune Evasion: JAK1 Downregulation and T-Cell Exhaustion Trends Enable Nodal Escape in Metastatic Canine HNSCC

**Figure 3:**
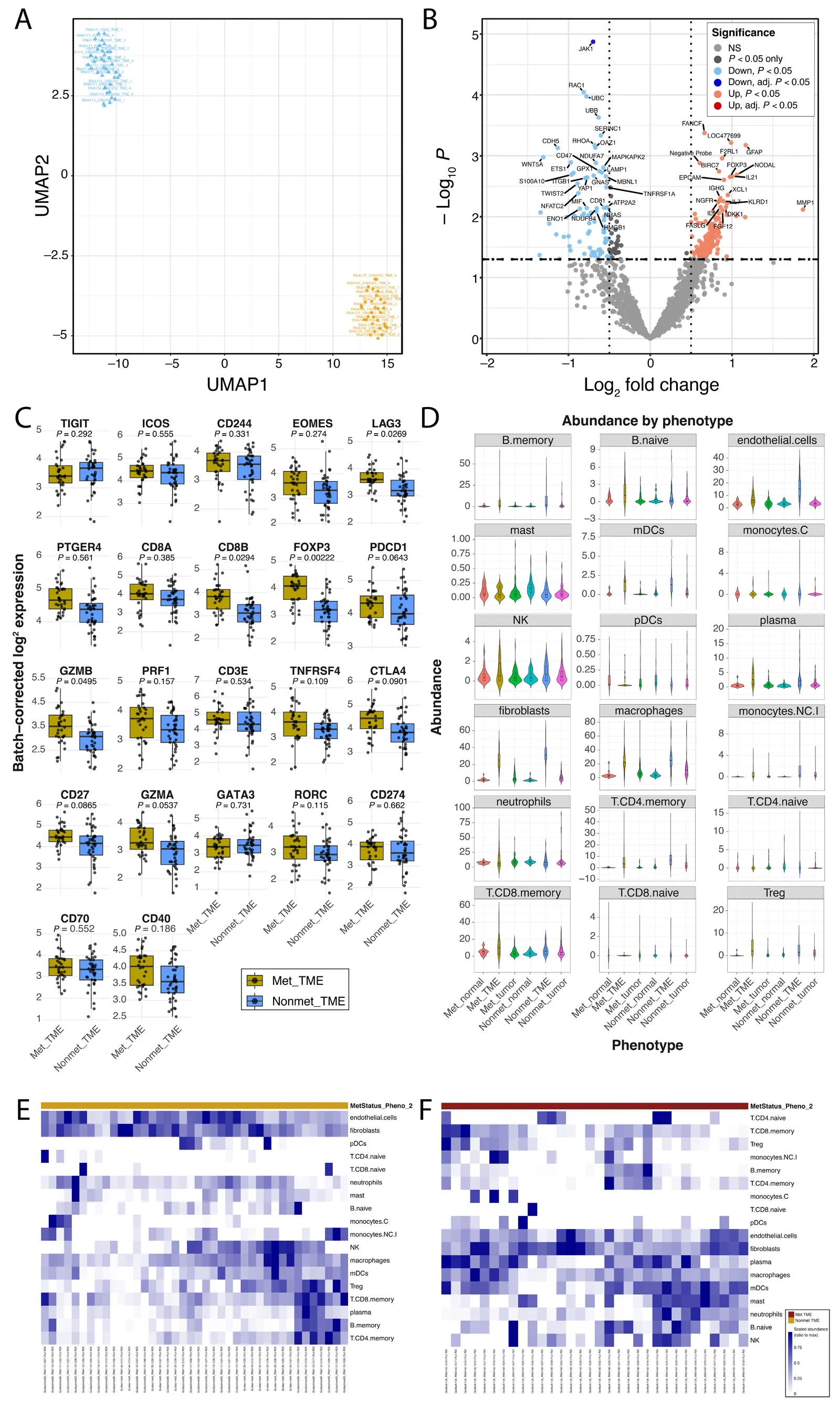
Differentially expressed genes and immune population between the TME of canine tumors with and without locoregional metastasis. (**A**) Distinct clustering of RNA transcripts between the TME in tumors with metastasis (yellow) and without (blue). (**B**) Volcano plot depicts the upregulated and downregulated differentially expressed genes in the TME (peritumoral stroma) in metastatic compared to nonmetastatic tumors. The log2 (fold change) and −log10 (*p* value) are indicated on the x- and y-axes. (**C**) Difference in total abundance of cell types in the TME, neoplastic tissue, and peritumoral normals of metastatic vs. nonmetastatic lesions. (**D**) Immune cell genes associated with T-cell exhaustion in the metastatic normal (red), TME (yellow), and tumor (green) compared to nonmetastatic normal (teal), TME (blue), and tumor (purple). (**E**,**F**) Patient variability in scaled abundance of immune composition in the TME in nonmetastatic (red) and metastatic tumors (yellow).

There were modest differences in the TME between tumors that did and did not metastasize. A total of 249 genes showed nominal differential expression (|log_2_FC| > 0.5, *p* < 0.05) with 173 upregulated and 76 downregulated. After FDR adjustment, only one gene retained significance, which was the downregulation of *JAK1* (Figure 3A,B). All FDR-non-significant findings are considered hypothesis-generating and require prospective validation.

The cellular components were further evaluated by performing mixed cell deconvolution analysis and direct comparison to assess key immune/inflammatory-associated genes (Figure 3C,D). Collectively, in the TME of tumors that metastasized (pN+) there was a non-significant trend toward increased mean abundance of T cells (CD8+ memory cells, T regs). Checkpoint molecules associated with T-cell exhaustion showed a consistent trend toward increased expression, including *LAG3* (*p* = 0.027), *PD1* (*p* = 0.064), and CTLA4 (*p* = 0.09), though only *LAG3* reached nominal significance among checkpoint inhibitors, and none survived FDR correction. *GZMB*, a cytotoxic effector, was also nominally increased (*p* = 0.050) but did not survive FDR correction. *CD274* (PD-L1) was not differentially expressed between pN+ and pN0 TME (log_2_FC 0.15, *p* = 0.66). Trends were maintained between pN+ and pN0 patients, but there was a high level of interpatient variability (Figure 3E,F).

Tumor-associated fibroblasts and macrophages were both markedly increased in the TME relative to normal tissue. Macrophage abundance did not differ between pN+ and pN0 TME (*p* = 0.61–0.65). Fibroblast abundance showed a nominal decrease in pN+ TME (*p* = 0.02). Among the 18 cell populations assessed, the only abundance difference to survive correction was a reduction in endothelial cell abundance in the pN+ TME (mean 7.5 vs. 17.6 in pN0).

GSEA (Hallmark collection) also identified functional differences in the pN+ TME, most notably the downregulation of hypoxia (NES = −2.54, FDR q < 0.001) and MYC target gene (NES = −2.44, FDR q < 0.001) signatures. TGF-beta signaling (NES = −2.08, FDR q = 0.004) and interferon-gamma response (NES = −1.57, FDR q = 0.045) were also downregulated, while the KRAS signaling-down signature (NES = 1.85, FDR q = 0.008) was upregulated.

### 2.3. Transcriptional and Immune Reprogramming of the Metastatic Lymph Node Niche: Upregulation of Keratins, a Pro-Inflammatory Myeloid Signature, and PD-L1 Separate Metastatic from Normal Lymph Node

**Figure 4:**
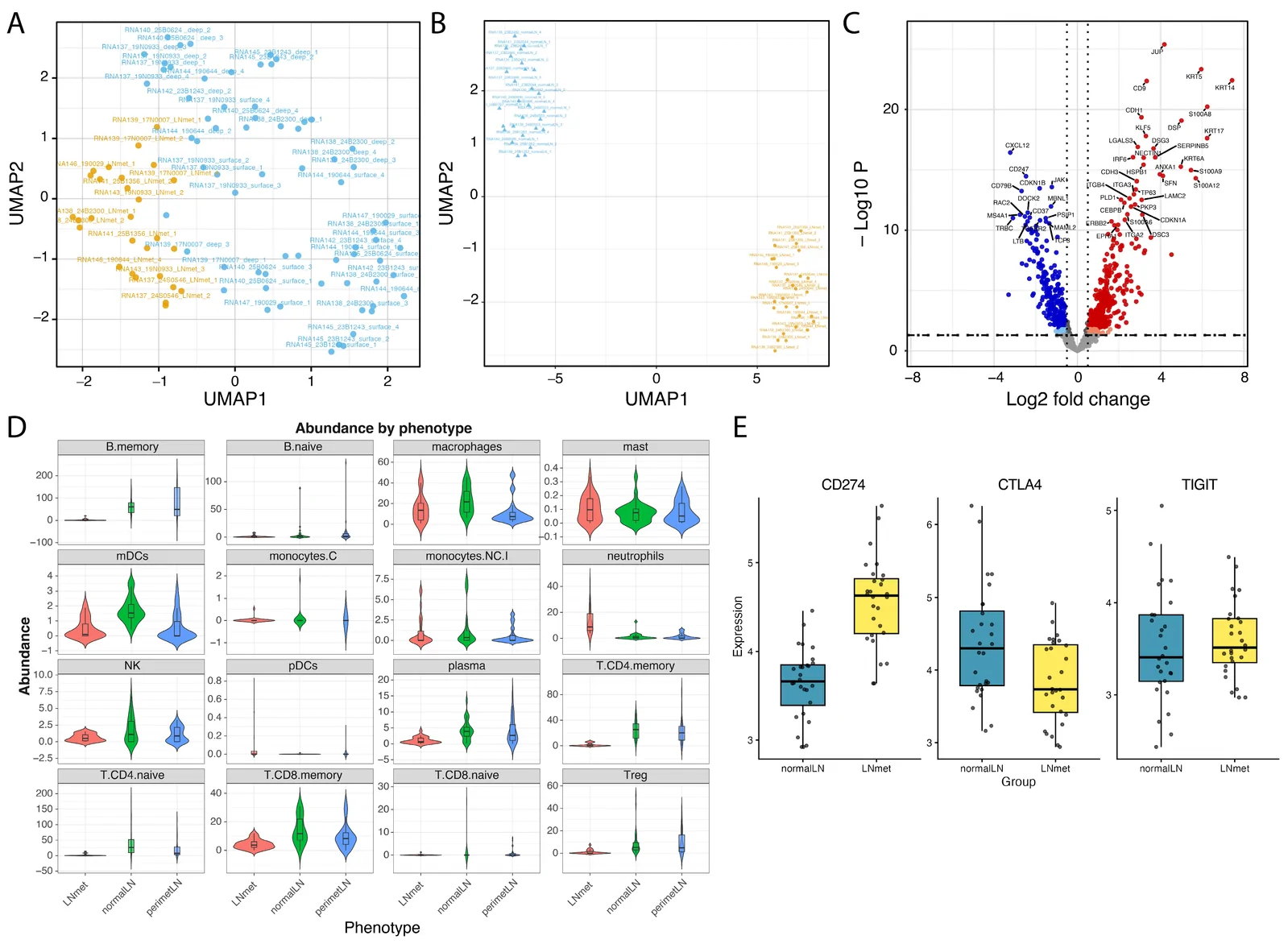
Molecular signatures of metastatic and nonmetastatic lymph nodes in dogs. (**A**) Overlap of gene expression between the metastatic niche in the lymph node (yellow) and the primary metastatic tumor (blue). (**B**) Distinct clustering of RNA transcripts between metastatic niche in the lymph node (yellow) and the normal lymph node (blue). (**C**) Volcano plot depicts the upregulated and downregulated differentially expressed genes in the metastatic compared to normal lymph nodes. The log_2_(fold change) and −log_10_(nominal *p* value) are indicated on the x and y-axes. (**D**) Difference in total abundance of cell types in metastatic (red) compared to peritumoral normal (blue) and normal (pN0; green) lymph node. (**E**) Differential gene expression of immune genes of interest in normal (blue) and metastatic (yellow) lymph nodes.

The next step was to compare the metastatic niche within the lymph node to the primary tumor and determine the molecular signature of the neoplasm when it establishes within a different organ (i.e., lymph node versus primary tumor). There was overlap in the gene expression between the metastatic lymph node and the primary tumor (Figure 4A). On initial comparison, there were 132 DEGSs (|log FC| > 0.5, *p* < 0.05) with 71 upregulated and 61 downregulated, yet after FDR adjustment, no genes remained significant.

Comparing the metastatic nodes to normal lymph nodes revealed distinct molecular signatures, with 754 DEGS (logFC > 0.5, nominal *p* < 0.05), with 605 retaining significance after FDR adjustment. The keratins were strongly upregulated, which fits with the presence of aberrant epithelial cells in the lymph nodes. A pro-inflammatory myeloid signature was also present. The increased mean abundance of neutrophils was accompanied by the upregulation of *S100A8*, *S100A9*, and *S100A12*, a combination associated with myeloid-derived suppressor cells [30]. There was also significant upregulation of *SPP1*, consistent with tumor-associated macrophage activity [31,32]. Concurrently, there was broad lymphoid depletion involving T-cell subsets (including Tregs) and memory B cells, accompanied by the downregulation of T-cell (*TRAC*, *TRBC*, *CD247*) and B-cell (*MS4A1*, *CD79A*, *CD79B*) transcripts. *CD274* (PD-L1) was upregulated (+0.91, q = 0.022), although other checkpoint markers including *PDCD1* (*PD1*), *LAG3*, and *TIGIT* were not significantly different (Figure 4B–E). 

Functionally, the metastatic region of the lymph node showed enrichment of pro-oncogenic gene sets relative to normal nodal tissue, including epithelial–mesenchymal transition, MTORC1 signaling, glycolysis, and hypoxia (all q < 0.01), along with C6 oncogenic signatures of EGFR and MEK activation (both q < 0.001). Compared directly to the primary tumor, gene set analysis revealed reduced hypoxia, oxidative phosphorylation, MTORC1, and glycolysis signatures in nodal deposits (q = 0.002–0.052), alongside enrichment of interferon alpha and gamma response, complement, and allograft rejection signatures (q < 0.002), reflecting the surrounding nodal immune environment.

### 2.4. The Pre-Metastatic Niche and “Soil” Preparation: Field Cancerization in Peritumoral Epithelium and Permissive Alterations in Metastasis-Free Lymph Node Regions

**Figure 5:**
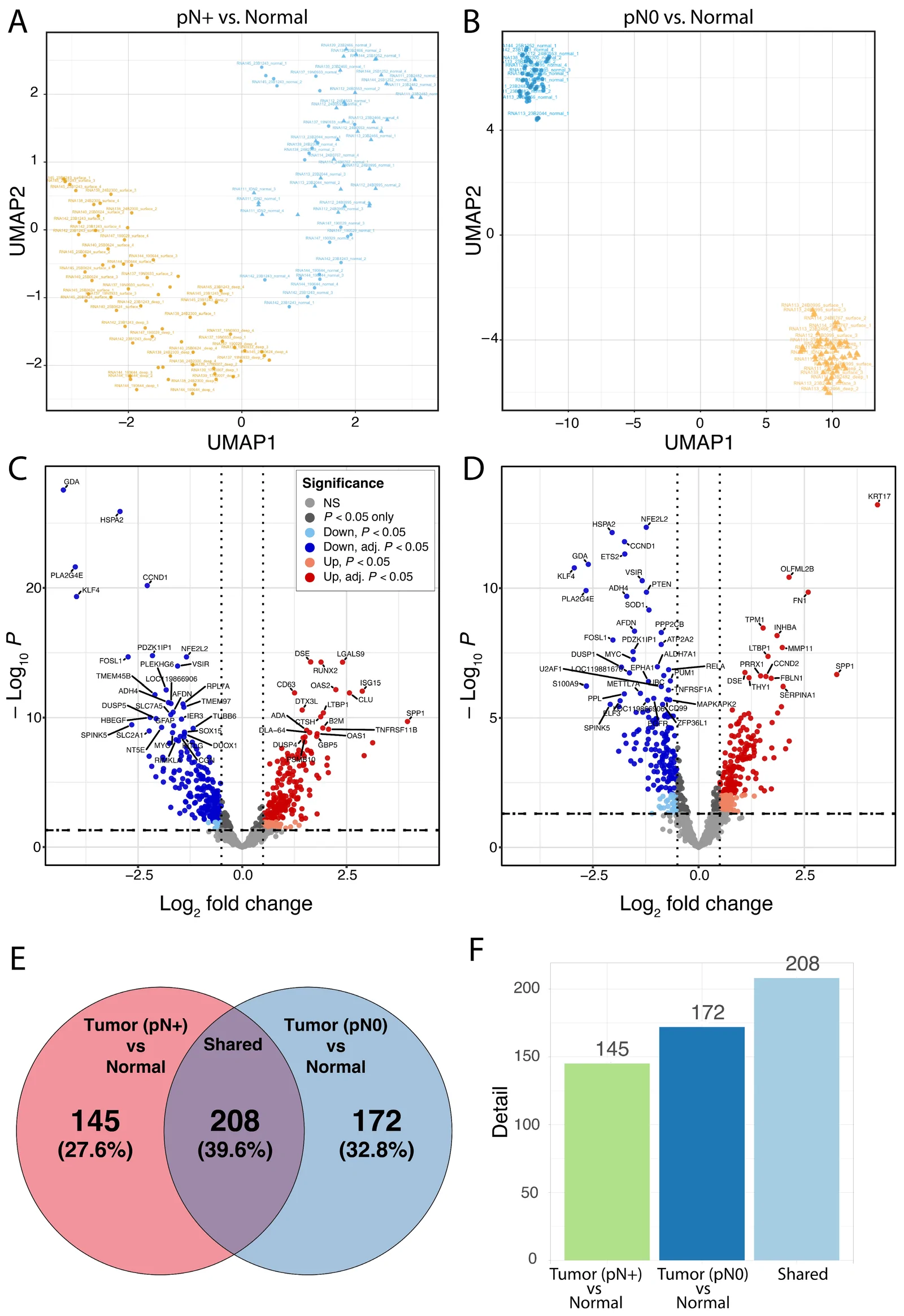
Differentially expressed genes between canine tumor epithelium and normal oral epithelial tissue. (**A**,**B**) Distinct clustering of RNA transcripts between tumor (yellow) and normal (blue) tissue on the UMAP. There is more distinct separation between the gene expression between nonmetastatic tumor and normal tissues (**B**). (**C**,**D**) Volcano plots depict the upregulated and downregulated differentially expressed genes from group comparisons between metastatic (**C**) or nonmetastatic tumors (**D**) and the corresponding patient matched normal tissue. The log2(fold change) and −log10(nominal *p* value) are indicated on the x- and y-axes. (**E**,**F**) Intersection analysis was conducted and the number of differentially expressed genes that overlap between metastatic tumors, nonmetastatic tumors, and normal phenotypes is depicted in the Venn diagram and corresponding bar chart.

To further explore the transcriptomic differences between pN0 and pN+ tumors, we then compared tumor tissue to normal tissue separately for each subtype. In both the pN0 and pN+ groups, UMAP visualization demonstrated the tissue-associated clustering of tumor and normal ROIs according to tissue type (i.e., on either side of 0 on the x-axis, Figure 5A,B), with visually more distinctive separation between the tumor and normal tissue ROIs in the pN0 group.

Differential expression analysis revealed 550 DEGS (log_2_fold change > 0.5, nominal *p* < 0.05) between pN+ HNSCC and normal tissue with 364 upregulated and 186 downregulated in the tumor. After FDR adjustment, 326 genes retained significance (171 upregulated, 155 downregulated). Among the most substantially upregulated genes were *KRT17*, *SPP1*, *FN1*, *COL1A1*, and *OLFML2B*. The most downregulated included *KLF4*, *PLA2G4E*, *S100A9*, *GDA*, and *SPINK5*. Comparing the pN0 tumoral epithelium to normal revealed 471 DEGS with 248 upregulated and 223 downregulated in the tumor. After FDR adjustment, 397 genes retained significance, with the most substantially upregulated genes including *SPP1*, *CXCL10*, *KRT17*, *ISG15*, and *CLU*. Conversely, the most downregulated genes included *GDA*, *PLA2G4E*, *KLF4*, *HSPA2*, and *FOSL1* (Figure 5C,D). Collectively there were 210 shared DEGS when comparing metastatic versus nonmetastatic tumors to normal (Figure 5E,F). All 210 changed in the same direction in both subtypes (117 downregulated, 93 upregulated in tumor), indicating a common tumor-versus-normal signature, regardless of metastatic status.

**Figure 6:**
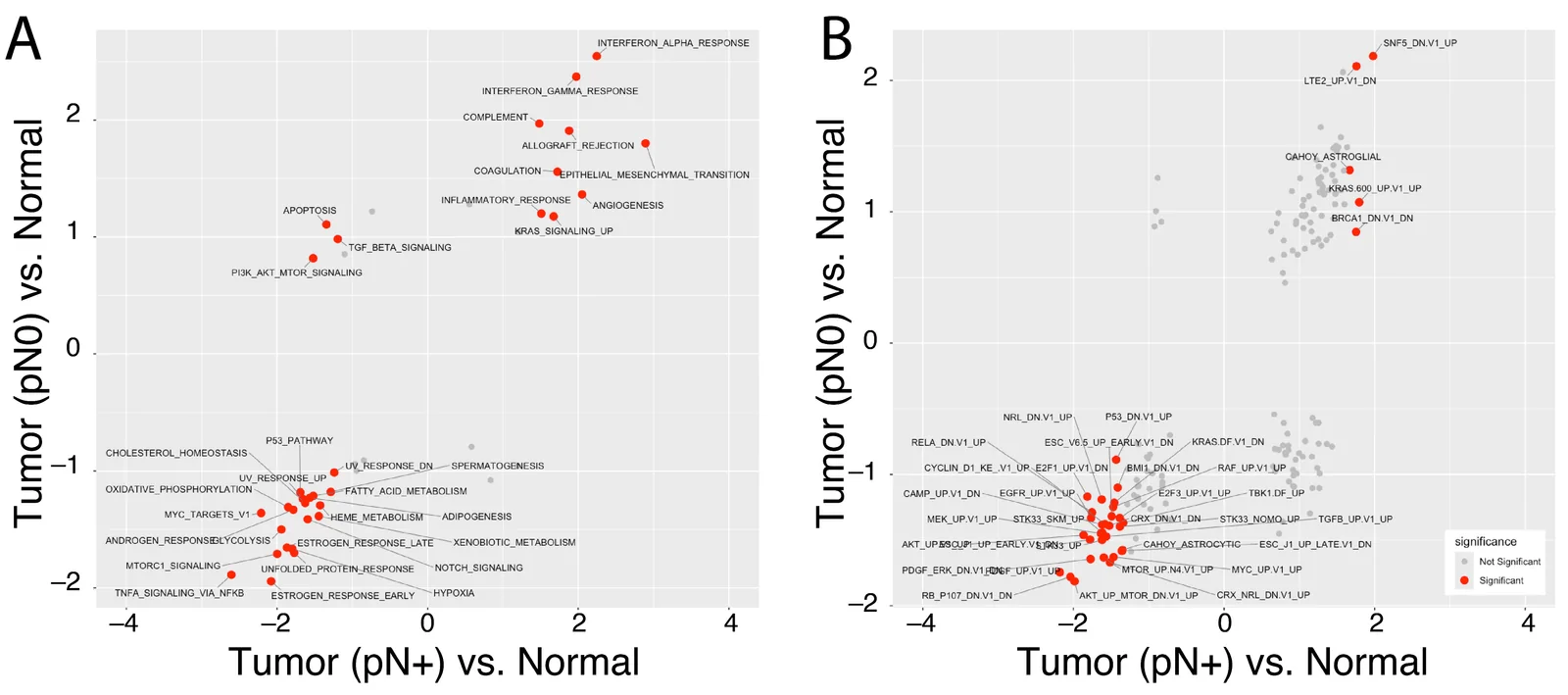
Functional enrichment analyses between canine tumor and normal tissue. Scatterplots depict normalized enrichment scores (NESs) from GSEA conducted with MSigDB Hallmark (**A**) and C6 oncogenic signature (**B**) gene sets across the comparisons of metastatic tumor to patient matched normal tissue and nonmetastatic to patient matched normal tissue. Gene sets exhibiting statistical differential enrichment or depletion (NES FDR q-value < 0.25) for the comparisons on the x-axes are shown in red.

From a functional standpoint, most upregulated and downregulated gene sets were shared when comparing nonmetastatic versus metastatic tumors to normal tissue (Figure 6A,B). Hallmark gene sets associated with PI3K-AKT-MTOR signaling, TGF-beta signaling, and apoptosis showed the most discordant directionality, with significant depletion in metastatic tumors and trending towards enrichment in nonmetastatic tumors.

Due to the unexpected clustering patterns (Figure 5A,B showing less distinctive tissue-associated clustering of normal and neoplastic tissue in pN+), we also compared the differential expression between normal tissues from each subset (pN0 vs. pN+). There were 714 DEGS based on nominal *p*-values, with 485 upregulated and 229 downregulated in the peritumoral normal tissue of the pN+ subset relative to the pN0 subset. Following FDR correction, 346 DEGS retained significance.

**Figure 7:**
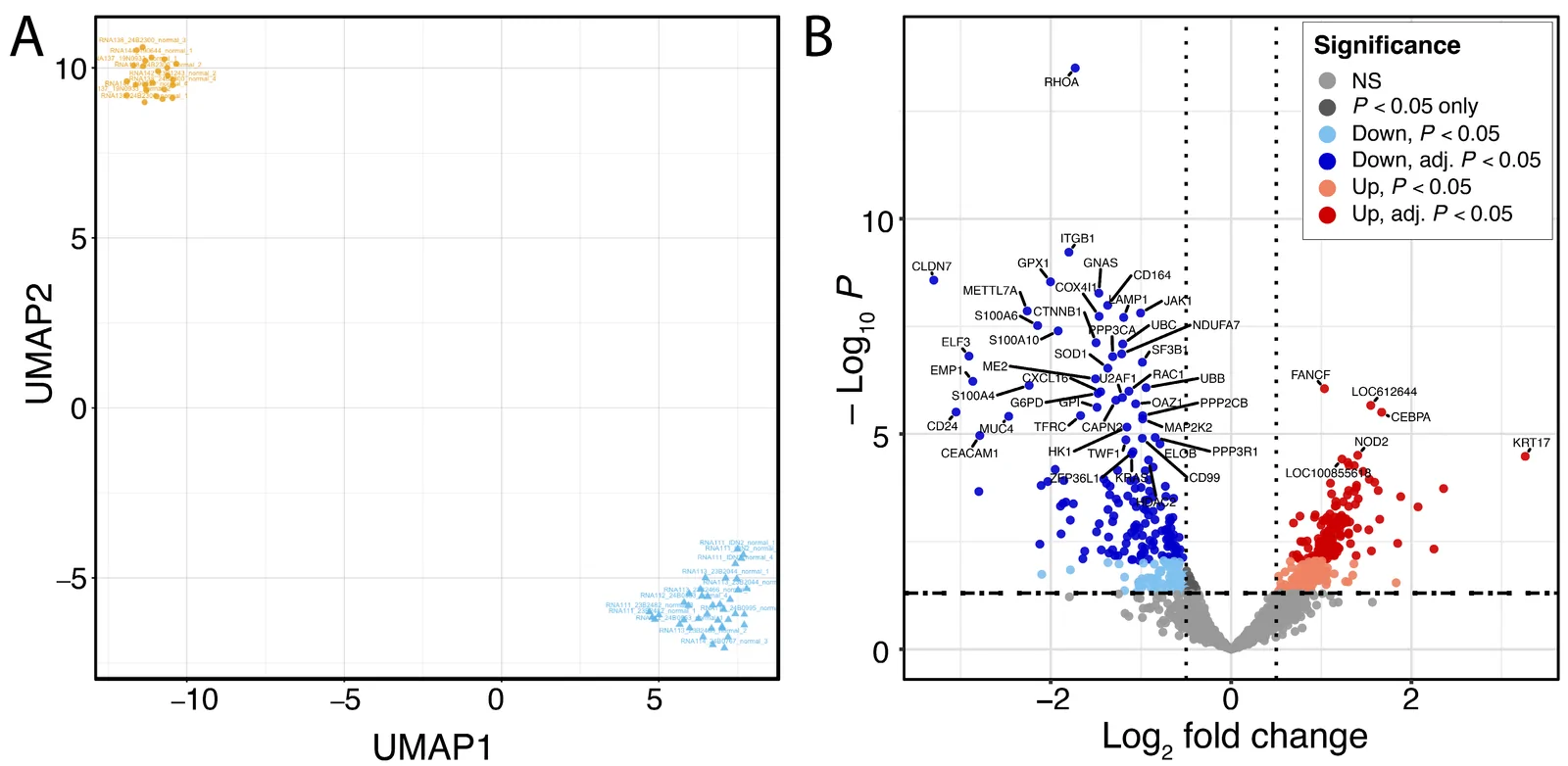
Differentially expressed genes between canine peritumoral normal epithelium in patients with and without locoregional metastasis. (**A**) Distinct clustering of RNA transcripts between normal oral tissue from metastatic patients (yellow) and nonmetastatic (blue) tissue on the uniform manifold approximation and projection. (**B**) Volcano plot depicts the upregulated and downregulated differentially expressed genes between normal tissue from the different patient subsets. The log2(fold change) and −log10(nominal *p* value) are indicated on the x- and y-axes.

Among the downregulated genes in the peritumoral normal epithelium of the pN+ patients were *CLDN7*, *CEACAM1*, *METTL7A*, and *SERPINB1*, each of which has been reported to lose expression during tumor progression. The calcium-binding proteins *S100A6*, *S100A8*, and *S100A9* were also reduced. The upregulated genes centered on squamous epithelial phenotype. *KRT17* and *LGALS7B*, markers of activated and squamous keratinocytes, were elevated alongside a coordinated set of terminal differentiation and cornified envelope genes including *KRT1*, *CALML5*, *FLG*, and *LOR*. Separately, *CCL20*, a chemokine implicated in tumor progression through immune cell recruitment, was upregulated. Together, these findings indicate that peritumoral epithelium from pN+ patients differs transcriptionally from that of pN0 patients, despite unremarkable histology (Figure 7).

Functionally, peritumoral normal tissue from pN+ versus pN0 cases revealed predominantly reduced pathway activity. These included broad downregulation of metabolic and biosynthetic programs, as well as p53, TGF-beta, and PI3K-AKT-MTOR signaling (q = 0.027, 0.002, and 0.031). The only enriched Hallmark sets were KRAS signaling (DN), comprising genes repressed by KRAS activity and therefore consistent with reduced KRAS pathway signaling, and allograft rejection (NES 1.77, q = 0.020).

Similarly, within the tumor-draining lymph node, we explored the molecular changes in the “normal” regions of metastatic lymph nodes (i.e., although metastasis was noted in other regions of the node, this region of lymph node appeared histopathologically normal). In comparing the normal peri-metastatic nodal tissue to completely normal nodes (no metastatic deposits in the node) 55 DEGS (log_2_FC > 0.5, nominal *p* < 0.05) were noted with 2 remaining significant after FDR correction. Both genes, *F13A1* and *DLA88*, were downregulated in the peri-metastatic nodal regions (log_2_FC −1.63 and −2.12, q = 0.016 for both). *DLA88* encodes a canine MHC class I molecule, and its reduced expression was also observed in the frankly metastatic regions of these nodes (log_2_FC −2.66, q < 0.001).

### 2.5. Validation of Our Results and Cross-Species Comparison

For validation of our transcriptomic findings qRT-PCR was conducted between metastatic and nonmetastatic tumors (pooled) for *PTEN*, *LOC477699*, and *JAK1* on the same FFPE blocks used for transcriptomic profiling. This confirmed our transcriptomic findings that *PTEN* was downregulated (fold change −1.49) and *LOC477699* was upregulated (fold change 1.75). *JAK1* was equivocal between metastatic and nonmetastatic tumors (fold change 1.01).

To further clarify the discriminatory power of our identified preliminary biomarkers (6 gene signatures), we performed an ROC analysis independently on the 14 dogs which had both lymph nodes and oral tissues available for tissue analysis. This analysis demonstrated the complete separation of pN0 and pN+ dogs within the study cohort, corresponding to an area under the curve (AUC) of 1.000 (Supplementary Figure S6). As the six genes were identified and evaluated using the same discovery cohort and that the cohort size was small, we consider this as an exploratory analysis, and independent validation will be required to determine the generatability of the signature prospectively.

We then compared our dataset to a historical HPV-negative (N = 15, Supplementary Table S1) GeoMx dataset [33], which we then stratified into pN0 and pN+ status. Between the human and canine GeoMX platforms, 1388 genes overlap and could be directly compared. Similar trends were seen between species, with 738 (53.1%) genes either both upregulated (535) or both downregulated (203) in the pN+ tumor compared to pN0 tumor ROIs. However, of the shared up- and downregulated DEGs between species, significance was only reached in 35 genes (34 human and 1 canine, none shared in both species).

**Figure 8:**
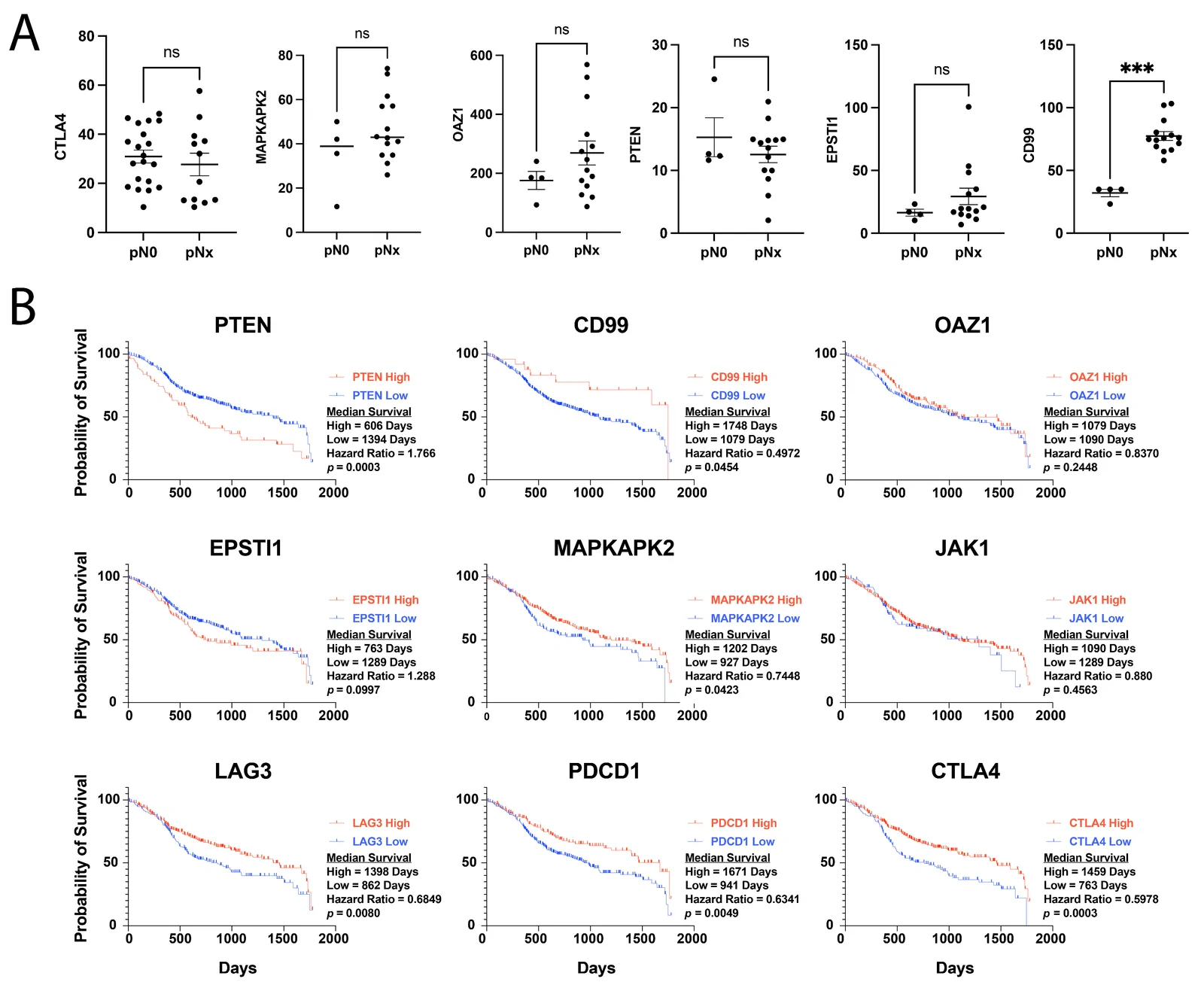
Selected genes of interest in human HNSCC. (**A**) Gene expression (Q3 normalized counts) in the human GeoMX samples revealing decreased median PTEN expression in pN+ (pNx) patients; ns: not significant, *** = *p* < 0.001. (**B**) Kaplan Meir Curves depicting gene expression association with 5-year survival based on the TCGA HNSCC dataset.

Of the tumor genes of interest identified in the initial canine analysis, *PTEN* was also downregulated in the human GeoMx dataset but did not reach nominal or adjusted significance. The findings for *EPST11*, *MAPKAPK2*, *OAZ1*, and *CD99* were discordant between humans and dogs. Only CD99 was significantly different between pN+ and pN0 tumors in the human GeoMx dataset (Figure 8A).

In the TME, 577 (41.5%) DEGs were concordant between the human and canine dataset, with 247 both upregulated in pN+ and 330 both downregulated in pN+. Of the shared DEGs, 13 genes were significantly different between pN+ and pN0 (human only). Of note, *JAK 1*, *CTLA4*, *LAG3*, and *PD1* were not significantly different between the pN+ and pN0 TME in the human GeoMx dataset, and the findings were discordant from the dogs. There was less separation between the TME of the pN0 and pN+ patients in the human GeoMX dataset (Supplementary Figure S7).

On a functional level, there were 42 shared pathways (nominal *p* < 0.05 in both species and concordant direction) between the canine and human pN+ tumor ROI and 8 shared pathways between the human and canine TME. In the pN0 tumors, there were 86 shared pathways in the tumor ROI and 46 shared in the TME (Supplemental Table S2). Of clinical note, tumorigenesis, multicancer invasiveness, KRAS, PI3k-MTOR, and collagen-degradation gene signatures were all upregulated among both human and cancer pN+ tumors.

For further clarification, we compared the key genes (*PTEN*, *EPSTI1*, *CD99*, *MAPKAPK2*, *OAZ1*, *LAG3*, *PD1*, *CTLA4*, *JAK1*) identified from our preliminary canine data to the TCGA head and neck repository. *PTEN* expression was lower in tumors with locoregional metastasis but this finding was not significant. *ESPT11*, *CD99*, and *OAZ1* had opposite trends to what was seen in dogs, but only *CD99* was significant. We also evaluated the effect of the genes of interest on 5-year survival. We identified that *CD99* (*p* < 0.05), *MAPKAPK2* (*p* < 0.05), *OAZ1* (*p* = 0.243), and *JAK1* (*p* = 0.456) were associated with better 5-year survival, consistent with our findings. Interestingly, *PTEN*, *PD1*, *LAG3*, and *CTLA4* were significantly associated with worse outcome (Figure 8B).

## 3. Discussion

This is the first study to describe the spatial transcriptomic landscape of canine HNSCC with and without pathologically confirmed locoregional metastasis (pN0 vs. pN+) resulting in high-level preliminary data on the mechanisms of locoregional metastasis and potential biomarkers for risk stratification. We identified six genes (*PTEN*, *EPSTI1*, *CD99*, *MAPKAPK2*, *OAZ1*, *LOC477699*) that may act as candidate biomarkers, with this signature resulting in complete separation between pN+ vs. pN0 patients on ROC analysis, and *LOC477699* upregulation and *PTEN* downregulation validated with QT-PCR. Further, we demonstrated the TME in metastatic tumors differed by downregulation of *JAK1* and a trend of T-cell exhaustion markers compared to nonmetastatic TME. Finally, the peritumoral normal epithelial tissue and lymph nodes in metastatic tumors appear to have evidence of field cancerization and permissive changes, respectively, which may indicate early disease (pre-malignant state).

Downregulation of *PTEN*, a tumor-suppressor gene, was one of the key differentiators between metastatic and nonmetastatic SCC within our cohort. *PTEN* downregulation primarily drives tumorigenesis through loss of the negative regulation of the *PI3K* pathway, increased epithelial–mesenchymal transformation, and promotion of cancer stem-cell behavior; yet, other impactful downstream protein interactions have also been implicated [34,35,36]. Complete genomic loss of *PTEN* in human HNSCC is rare [37], yet decreased levels of *PTEN* protein have been reported in up to 30% of samples [38] and have been associated with both a worse prognosis and drug resistance [39,40]. Despite the implications that *PTEN* loss results in more aggressive biologic behavior in HNSCC, to the authors knowledge, there is no documented direct link between *PTEN* activity and presence of locoregional nodal spread. This association, however, has been well documented with breast carcinoma [41,42,43], highlighting that this is likely also an importer driver in locoregional metastasis of oral carcinoma, and likely a key biomarker for risk stratification. This suspicion was strengthened by comparison with the human GeoMx dataset and the TCGA, where similar trends were noted with *PTEN* downregulation being associated with pN+ status, although significance was not reached. Conflicting findings were found when evaluating the effect of downregulation of *PTEN* on five-year survival, although survival is impacted by other factors, not only locoregional metastasis status. Further evaluation and validation of the potential of *PTEN* to be utilized as a biomarker of locoregional metastasis across species is warranted.

The other genes that discriminated the pN+ from the pN0 tumors in the canine tumors included *OAZ1*, *LOC477699*, *EPSTI1*, *MAPKAPK2*, and *CD99*. *OAZ1* is also a tumor-suppressor gene, which inhibits cell proliferation and promotes terminal cell differentiation. It has been shown to be downregulated in numerous human oral cancer cell lines [44]. Further, one study showed that *OAZ1* expression induced the conversion of a SCC cell line to a less metastatic subset [45], suggesting it may be a promising cross-species biomarker of metastatic potential. In the human GeoMx and TCGA datasets, no significant difference was seen in expression with nodal status. Yet, similar trends were noted when focusing on 5-year survival, with high CD99, *MAPKAPK2* and *OAZ1* expression being associated with better 5-year survival. The role of *LOC477699*, *ESPTI1*, and *CD99* have not been fully elucidated in dogs, but are associated with markers of matrix remodeling and metastasis, macrophage function, and immune cell adhesion and migration, respectively [46]. *LOC477699* is the canine ortholog for *alpha-2 macroglobulin* (A2M), which has been reported to promote invasion, migration, and EMT in certain human malignancies [47,48]. Due to the strong upregulation in our preliminary dataset, further investigation into the role of A2M in EMT and disease progression in human head and neck squamous cell carcinoma is warranted.

From a functional standpoint, gene sets associated with PTEN loss showed nominal enrichment in metastatic tumors but did not survive FDR correction and should be regarded as preliminary. The most robust functional finding was reduced interferon alpha and gamma response signatures in metastatic tumors, consistent with the reduced *JAK1* expression observed in the pN+ TME. *JAK-1* is a significant player in immune regulation through the JAK-STAT pathways. Although upregulation, especially of STAT-3, has been previously implicated in progression of HNSCC [49,50], conflicting evidence is emerging for the role of JAKs and the JAK-STAT pathways in immune surveillance of neoplastic tissue [51,52]. *JAK1* is required for signaling through both type I and type II interferon receptors, and loss-of-function *JAK1* alterations are an established mechanism of resistance to immune checkpoint blockade in human cancers [53]. This offers a plausible mechanistic link between reduced *JAK1* expression, the concurrent downregulation of the interferon-gamma response gene set, and the lack of PD-L1 induction observed in the pN+ TME. Of further note was the seemingly discordant decrease in both endothelial cell abundance and the hypoxia signature in pN+ TME. The nominal increase in GZMB expression in pN+ TME may be mechanistically relevant to the reduced endothelial cell abundance observed, as extracellular GZMB degrades the extracellular matrix and disrupts endothelial adhesion [54]. Whether these processes also contribute to the discordant reduction in the hypoxia signature in pN+ TME should be further explored.

Increased *PDL1* was also seen in the metastatic niche within the lymph node compared to normal nodal tissue. This mirrors the clinical picture in human HNSCC, where signs of T-cell exhaustion are seen with progressive disease, and there is upregulation of the PDL:PD-L1 ligand: receptor activity in lymph nodes with extranodal extension, playing a pivotal role in the immunosuppressive environment and allowing for nodal metastasis [55,56,57]. This suggests that there is a subset of canine patients with SCC that may benefit from immune checkpoint inhibition to allow T cell expansion and differentiation from the tumor-draining lymph node, and there may be a role in neoadjuvant or adjuvant therapy to prevent locoregional metastasis.

Comparison of our canine spatial transcriptomic data with human data showed conserved trends across species: most genes shifted in the same direction and shared functional changes with locoregional metastatic status (pN0 vs. pN+). However, among the candidate biomarkers identified from the canine data, only the downregulation of *PTEN* was concordant between species, while *MAPKAPK2*, *EPSTI1*, *CD99* and *OAZ1* moved in the opposite direction (decreased in dogs, increased in human PN+).

These discordances may reflect biologically meaningful cross-species differences, particularly for *MAPKAPK2* and *EPSTI1*. In human HNSCC, upregulation of *MAPKAPK2* is consistently pro-tumorigenic and pro-metastatic, acting through post-transcriptional control of mRNA stability for targets including *p27*, *MKP-1*, *TNF-α*, *VEGF*, *IGFBP2*, *MUC4*, *PRKAR2B* [58,59]. Its activity was recently shown to be critical for lymph node and lung metastasis in HPV-negative HNSCC, where silencing reduced migration, invasion and metastasis in an orthotopic mouse model. No data exist on its oncogenic role in dogs in any tumor type for comparison, but findings from this study suggest the activity of *MAPKAPK2* in canine HNSCC may represent a key biologic difference to consider in translational work.

*EPSTI1* likely also differs mechanistically between species. Most data come from human breast carcinoma, where upregulation clearly promotes EMT, invasion and metastasis [60,61,62,63]. To our knowledge, only one study has examined the role of upregulation in HNSCC: in oral SCC lines, knockdown of *EPSTI1* suppressed proliferation and reversed EMT, whereas in a lung carcinoma model, knockdown had the opposite, protective effect, informing us that its role is tumor-type dependent [64]. In colon cancer, higher *EPSTI1* is also associated with better prognosis, higher immune scores (especially M1 macrophage activity), and IFN and JAK-STAT pathways [65]. A similar role is plausible in canine HNSCC, where downregulation of *EPSTI1* may alter the tumor microenvironment, resulting in increased metastatic potential. This is consistent with the broader interferon suppression and decreased *JAK1* in pN+ tumors in dogs, which occurred without a significant difference in EMT activity.

*OAZ1* and *CD99* were also discordant (downregulated in dogs, upregulated in human pN+), yet TCGA 5-year survival data matched our canine findings (downregulation results in poorer survival), and limited literature supports lower expression of both genes in HNSCC. *CD99* is altered in both directions across malignancies [66], with pan-cancer analysis showing significant downregulation in head and neck cancer [67], though its protective mechanism remains unclear. *OAZ1* limits polyamine synthesis and promotes terminal cell differentiation, so higher expression is functionally tumor suppressive; in the SCC15 oral SCC line, increased *OAZ1* inhibited proliferation and induced differentiation, consistent with the canine result. The *OAZ1* and *CD99* discordance (downregulation in dogs, upregulation in human pN+) may therefore reflect small sample size and ROI selection rather than true species differences, particularly since TCGA data agreed with the canine data. Conversely, the differences between the GeoMx data and TCGA data may reflect the difference in analysis (tumor-specific vs. bulk analysis, respectively) and the GeoMx data displaying species discordance may be a true biologic discordance. Future tumor-specific spatial transcriptomic or single-cell analysis with expanded sample sizes in both dogs and humans are needed.

In humans, non-invasive salivary biomarkers have also emerged as an important complementary approach to tissue-based diagnosis in OSCC. An early study identified reduced salivary miR-125a and miR-200a in OSCC patients compared with healthy controls, among the first demonstrations that saliva-based microRNAs could serve as biomarkers for oral cancer detection [68]. More recently, a discovery-and-validation study identified an eight-miRNA salivary signature that distinguished oral cancer from healthy controls with high diagnostic accuracy (AUC 0.954), with related four-miRNA panels discriminating between oral cancer and oral potentially malignant disorders (OMPDs) (AUC 0.911) and OMPD from controls (AUC 0.807) [69]. Beyond microRNAs, the salivary non-coding RNA repertoire now extends to long non-coding RNAs whose levels track with tumor stage, nodal status and surgical resection (i.e., the presence of tumor) [70]. These salivary RNA signatures represent a complementary, minimally invasive biomarker modality to the tissue-based transcriptomic candidates identified in the present study. Whether any of the tissue biomarkers identified here, such as PTEN, correlate with circulating or salivary non-coding RNAs implicated in human OSCC remains to be determined and represents a valuable direction for future work bridging tissue-based and liquid biopsy approaches to biomarker discovery in both species.

The metastatic nodal deposits also had a strong pro-inflammatory signature compared to normal nodal tissue, with increased neutrophils and tumor-associated macrophage activity (*SPP1*) [31,32]. Increased SPP1 was also seen in both metastatic and nonmetastatic tumor compared to normal, underpinning the importance of this gene as a driver of carcinogenesis both locally and at distant sites. This highlights another impactful cross-species similarity as SSP1 has been identified as a key biomarker in human HNSCC [71,72]. Comparing the metastatic niche directly to the primary tumor, no genes retained significance after FDR adjustment. This comparison was limited by the small number of patients contributing nodal metastases, and larger cohorts will be needed to determine whether meaningful transcriptional differences exist between primary and nodal disease.

Lastly, a surprising finding in this study was that there were distinct differences in the molecular signature of histologically normal tissues adjacent to tumors with and without locoregional metastasis, suggesting there may be field cancerization occurring in the normal tissue next to more aggressive lesions. Within the peritumoral normal tissue in pN+ patients, several genes previously implicated in tumor progression were downregulated, including *CLDN7*, *CEACAM1*, *METTL7A*, and *SERPINB1* [73,74,75,76]. Concurrently, there was upregulation of stress keratin *KRT17* and *LGALS7B*, alongside a coordinated set of terminal keratinocyte differentiation and cornified envelope genes (*KRT1*, *CALML5*, *FLG*, *LOR*), and of the chemokine *CCL20.* Together, these changes suggest that the squamous differentiation program is already perturbed in tissue that appears histologically unremarkable. Notably, the downregulated genes and the stress keratins showed concordant changes in the metastatic tumors themselves, suggesting overlap in transcriptomic activity between the tumor and its adjacent “normal” epithelium. Similarly, the peri-metastatic lymph node showed changes potentially permissive to metastasis, including downregulation of *F13A1* and *DLA88*; the latter encodes a canine MHC class I molecule, and its reduced expression extended into the frankly metastatic regions of these nodes. These genes may represent molecular testing opportunities for detecting and intervening in pre-metastatic states.

The limitations of this work include the small biologic sample size (n = 18 dogs, 14 with matched oral and nodal tissue available for analysis), which may have biased results and led to the overinterpretation of results, especially regarding the TME, which is known to be highly heterogeneous. This is especially notable given that the tumors in the pN0 and pN+ groups differed in size, location, and pathologic features, which may have influenced their transcriptomic signatures and confounded our findings. Given our already small sample size, stratifying by these tumor features was not realistic; this limitation must therefore be acknowledged as a potential confounder, independent of metastatic status alone. We used a historical dataset which can introduce batch effect; yet we included anchor samples that were run in both datasets to ensure we could correct for any anomalies and felt comfortable that a large bias was not introduced (Supplementary Figures S1–S5). Further, while we explored the discriminatory power of our identified gene signature with ROC analysis applied independently to all samples, the six genes were identified and evaluated using the same discovery cohort, this thus should be considered exploratory only. We validated our transcriptomic findings by qPCR using canine-specific primers on the same FFPE blocks used for transcriptomic analysis, but we did this on select genes only (*PTEN*, *LOC477699*, *JAK1)* and did not perform protein-level validation by immunohistochemistry. Canine-validated IHC antibodies are not currently established for most of the genes of interest in canine tissue and rigorous species-specific antibody validation was beyond the scope of this study. Future work incorporating IHC would further strengthen confidence that transcriptional changes are reflected at the protein level. Despite these limitations, this study reveals distinct molecular signatures of tumors with and without locoregional metastasis, as well as relevant findings from other peritumoral regions between these two cohorts that may directly diagnostic and therapeutic interventions between pN0 and N+ patients.

Building on these preliminary findings in dogs and their parallels in human OSCC, we propose a three-component risk score to prospectively guide decisions about elective neck dissection. The score could be implemented as a single multiplexed assay applicable across species and would integrate the following: (1) tumoral *PTEN* expression; (2) a microenvironmental signature combining *JAK1* expression with an immune exhaustion score, using *LAG3* and *CTLA4* as surrogate markers; and (3) *KRT17* upregulation in the peritumoral adjacent normal epithelium. Prospective validation will be needed to establish cut-offs and to determine whether the full tumoral six-gene signature outperforms *PTEN* alone, but we view this as a logical next step toward clinical translation.

## 4. Materials and Methods

### 4.1. Clinical Samples

This study evaluated 31 formalin-fixed paraffin-embedded specimens from 18 dogs (14 with matched nodal and oral samples for analysis) with naturally occurring HNSCC that were surgically excised with concurrent excision of the lymph node confirming a pathologically N0 (n = 10) or N+ (n = 8) status. Samples were obtained from clinical patients conducted upon institutional animal care and use protocol review and approval (UC-Davis IACUC #23110) or from the necropsy archives at UC-Davis. Patient characteristics are shown in Table 1.

### 4.2. Spatial Transcriptomics

The spatial transcriptomic analysis of nonmetastatic and metastatic canine head and neck squamous cell carcinoma (HNSCC) specimens was performed on the NanoString GeoMx Digital Spatial Profiler (DSP) platform [65] using the GeoMx Canine Cancer Atlas assay to assess the expression of 1962 genes and 48 control targets. A review of the hematoxylin and eosin (H + E)-stained slides was conducted by board-certified pathologists (NVA, B.G.M, CL) to choose the most appropriate block per case. Unstained tissue sections (5 µm) were then cut from the corresponding FFPE blocks and mounted on Superfrost slides (Thermo Fisher, Waltham, MA, USA). GeoMx DSP analysis was performed by the Genomics Shared Resource (Department of Pathology and Laboratory Medicine, University of California, Davis School of Medicine) according to NanoString’s standard protocol for NGS readout and as previously described [28]. The slide-preparation steps were conducted on a Leica BOND RX stainer, followed by in situ hybridization of the GeoMx Canine Cancer Atlas probe set, and tissue sections were stained with fluor-conjugated morphology marker antibodies for pan-cytokeratin (PanCK; Alexa 532, Cy3/568 nm channel), CD45 (Alexa 594, Texas Red/615 nm channel), CD3 (Alexa 647, Cy5/666 nm channel), and the SYTO-13 nuclear stain (FITC/525 nm channel, for instrument focus).

**Figure 9:**
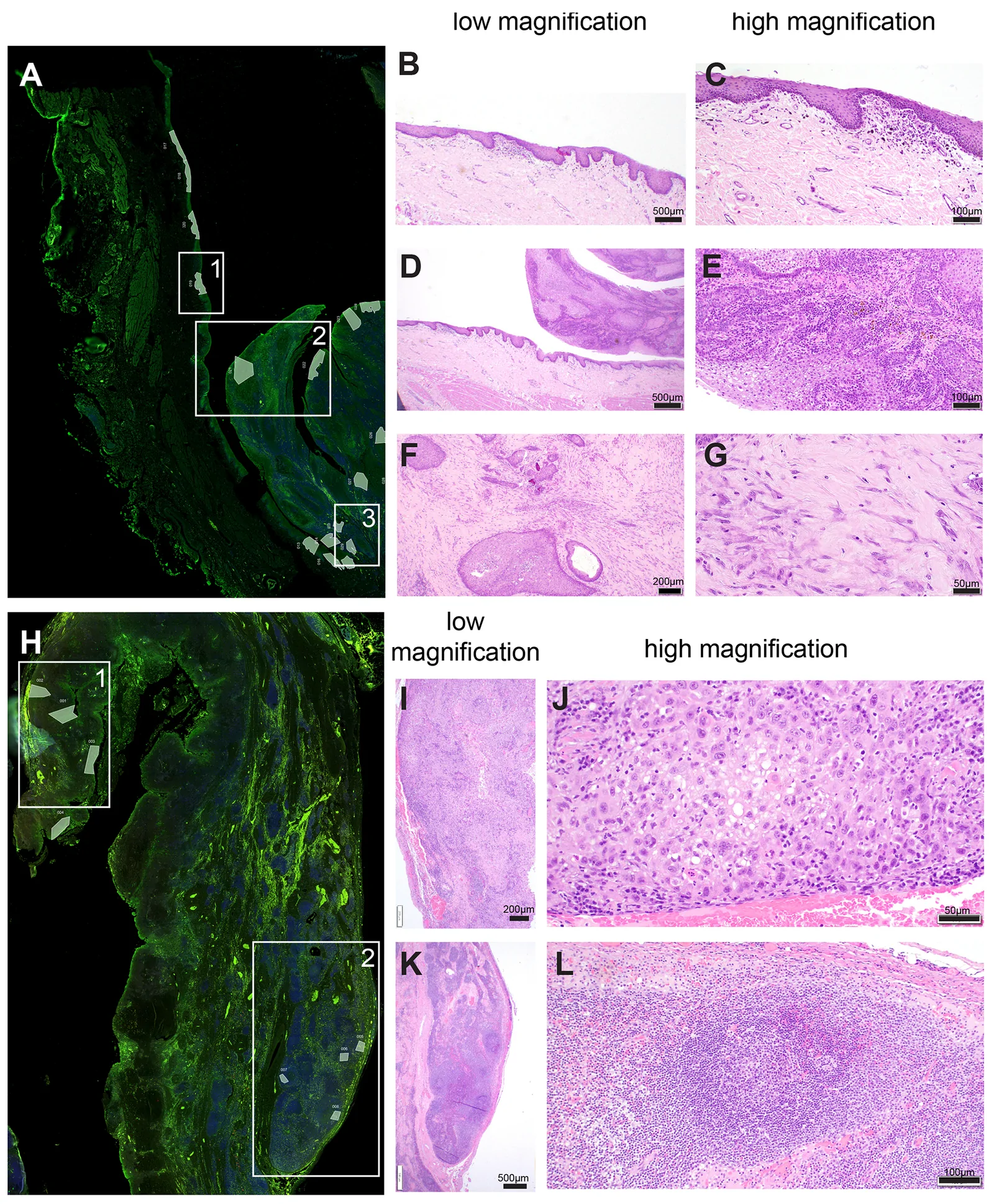
Immunofluorescent and hematoxylin and eosin (H&E) photomicrographs representing the region of interest (ROI) selection approach. (**A**) Immunofluorescent 1− capture of a primary canine HNSCC with attached section of histologically normal oral epithelium. Areas enclosed in white rectangles 1, 2, and 3 are shown with lower and higher magnification on H&E-stained tissue in (**B**–**G**). (**B**) Normal oral epithelium area corresponding to a white rectangle #1 H&E, Bar = 500 µm. (**C**) Higher magnification of B capture. Note minimal lymphocytic inflammation and melanin-laden macrophages in the subepithelial stroma. H&E, Bar = 100 µm. (**D**) Low magnification of tumor area, corresponding to white rectangle #2, H&E, Bar = 500 µm. (**E**) Higher magnification of (**D**), note disorganized and invasive growth of neoplastic epithelial cells with irregular trabeculae of epithelial cell extending into the subepithelial stroma without apparent alignment to the basement membrane and associated lymphocytic inflammation H&E, Bar = 100 µm. (**F**) Low magnification of tumor microenvironment area, corresponding to white rectangle #3 highlighting mesenchyme composed of spindle cell population beaded with small areas of invasive neoplastic islands H&E, Bar = 200 µm. (**G**) High magnification of the peritumoral mesenchyme with reactive, plump fibroblast supported by collagenous extracellular matrix H&E, Bar = 50 µm. (**H**) Immunofluorescent 1x capture of a canine HNSCC that metastasized into regional lymph node. Areas enclosed in white rectangles 1 and 2 are shown with lower and higher magnification on H&E-stained tissue in (**I**–**L**). (**I**) Low magnification of area in the white rectangle #1. Note effacement of normal lymph node architecture by densely cellular neoplastic cells arranged in sheets and irregularly outlined, coalescing islands H&E, Bar = 200 µm. (**J**) High magnification of (**I**) capture. Note there is no evidence of lymph node tissue, which is replaced by sheets of highly pleomorphic, variably degenerate neoplastic epithelial cells with various degrees of keratinization. Lymphocytes infiltrate between the neoplastic cells H&E, Bar = 50 µm. (**K**) Low magnification of area in the white rectangle # 2. Note the more-or-less-preserved lymph node architecture with prominent lymphoid follicles and medullary sinuses draining hemorrhage H&E, Bar = 500 µm. (**L**) High magnification of (**K**), depicting subcapsular lymph node sinus subtended by a lymphoid follicle H&E, Bar = 100 µm.

Slides were scanned at 20X magnification and 4 regions of interest (ROIs) for each specific tissue phenotype were selected for analysis. Tissue types for the primary tumor samples included normal surface epithelium, carcinoma tumor tissue, and tumor microenvironment (TME) (peritumoral mesenchymal stromal tissue infiltrated with CD45+ cells). For the normal lymph nodes (pN0), tissue types included normal tissue architecture (medullary sinus, subcapsular sinus; 2 ROIs from each). For metastatic nodes (pN+), tissue types included the metastatic deposit in the node, and when available, “normal” nodal tissue around the metastatic deposit (Figure 9).

**Figure 10:**
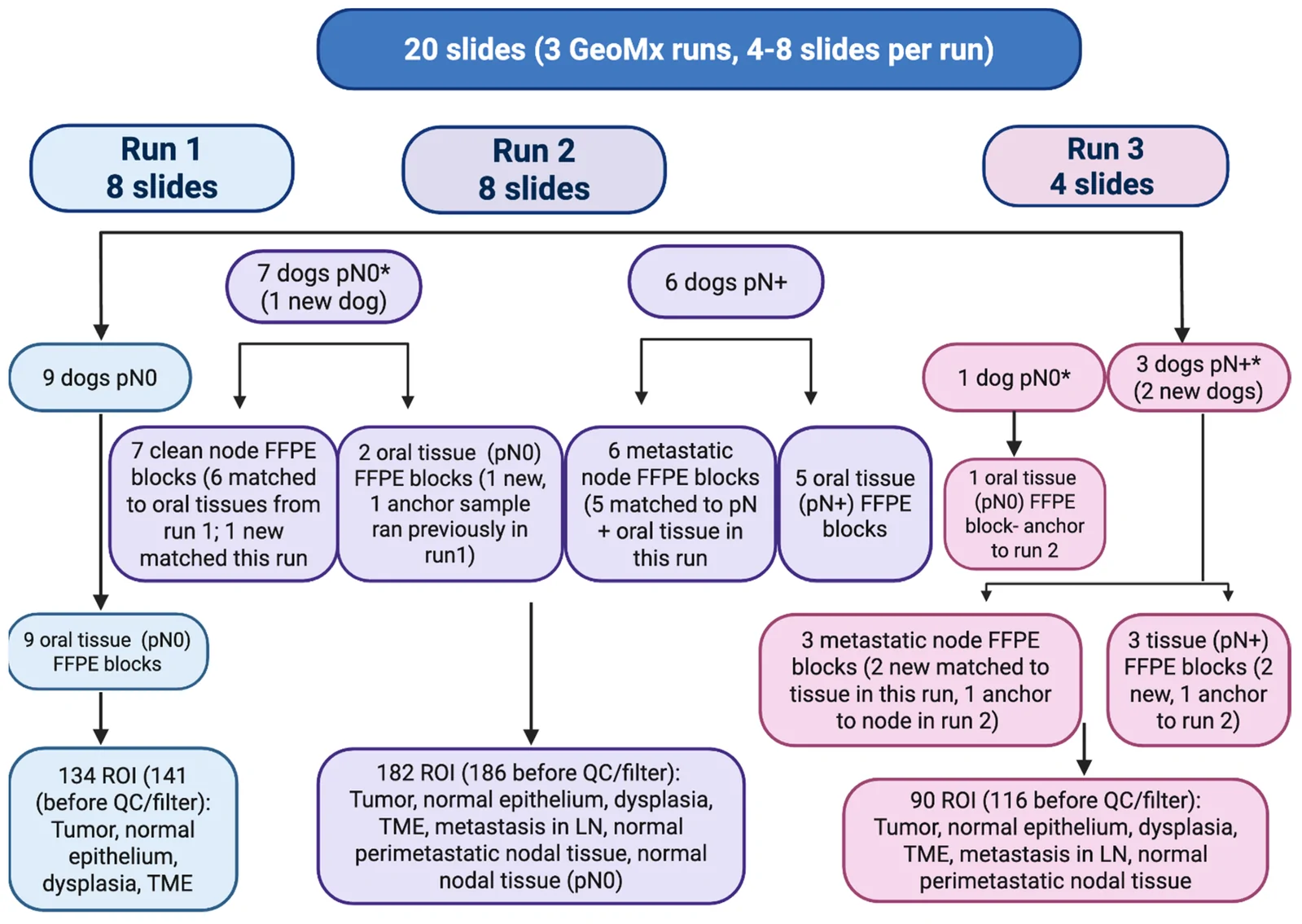
Breakdown of transcriptomic samples utilized within the study from 10 pN0 dogs and 8 pN+ dogs. The ROI were collected from 3 separate runs, with anchor samples included to link between runs 1–2 and 2–3. Oral tissues and lymph nodes were occasionally run in separate runs. Note: * represents dogs that had been previously utilized in other runs—either split of lymph nodes and oral tissues among runs, or anchor samples.

This study involved data acquired from 20 slides from separate runs conducted on the same GeoMx DSP instrument. Spatial profiling of the nonmetastatic primary tumors (n = 9) was reported previously [28] and the raw transcriptomic count data was integrated in the current study for new analyses. Metastatic primary tumors, lymph node metastases, and normal tissues (epithelial, lymph node) were analyzed across two new runs (Figure 10). To ensure that any batch effects were remediated, selected patient samples (i.e., anchor samples; multiple ROIs from several tissue phenotypes per sample, including pN0 nonmetastatic primary tumors) were either re-run or replicated across the current analysis runs (Figure 10, Supplementary Figure S8, Supplementary Tables S3 and S4).

In total, 406 non-segmented ROIs were selected. ROI aspirates were collected following UV photocleavage and ROI-specific indexed libraries were prepared from the DSP DNA oligonucleotides using high-fidelity PCR with GeoMx SeqCode Illumina-compatible primers. Validated libraries (i.e., having expected amplicon size of 162 bp) were sequenced on an NovaSeq X Plus Sequencing System (Illumina, San Diego, CA, USA) using paired-end sequencing with unique dual index (UDI) reads (2 × 150 bp paired-end, i7/i5 reads = 8 bp) and with a target sequencing depth factor of 100 paired-end reads/mm^2^ area collected.

### 4.3. Data Analysis

Raw FASTQ sequence data was converted to digital counts (DCC files) using the GeoMx NGS Pipeline (Bruker Spatial Biology, Bothell, WA, USA) and initial analyses were conducted with the GeoMx DSP Data Analysis Suite (Bruker Spatial Biology, Bothell, WA, USA). GeoMx NGS pipeline quality and sequencing metrics, including raw, trimmed, stitched, aligned, and deduplicated read counts per ROI, are summarized in Supplementary Table S3. Quality control (QC) parameters were assessed, and underperforming ROIs/segments were flagged based on the following criteria: raw read threshold of <1000 reads, <80% read alignment, <50% sequencing saturation, negative probe count geomean <10, No Template Control (NTC) count >1000, nuclei count <50, and surface area <1600 mm^2^. Biological probe QC was conducted to identify and exclude low outlier probes that had average counts across all segments of ≤10% of the counts for all probes to a particular gene and/or global outliers that failed the Grubbs outlier test in ≥20 segments. The dataset was then filtered to remove segments with exceptionally low signal of <10% panel target genes detected above the expression limit of quantitation (LOQ; calculated at a confidence threshold of 2 standard deviations above the geomean of the negative probes). A total of 406 ROIs passed QC filtering and were included in the downstream analyses.

The filtered dataset and ROI annotations were imported into the R statistical environment (version 4.5.2) using the read.delim function. Raw probe counts were globally normalized using third quartile (Q3) normalization and subsequently log_2_-transformed prior to downstream analysis and visualizations. Batch effects were removed from the expression matrix using the removeBatchEffect function from the limma package with slide (scanID) as the batch variable while preserving biological variation. Batch effects due to processing the samples across three DSP runs were not prominent prior to batch correction and were minimized following application of batch correction (Supplementary Figures S1–S5). Dimensionality reduction analyses of batch-corrected expression data were conducted with principal components analysis (PCA; R prcomp function) and uniform manifold approximation and projection (UMAP; uwot package version 0.2.4). Differential expression analysis was performed using the limma package (version 3.66.0) [77]. Individual specimens (i.e., dogs, specific tissues) were considered independent biological units whereas ROIs represented distinct spatial observations nested within each specimen. Linear models were fitted to log2-transformed, normalized expression data, with the design matrix including the biological group of interest and slide (ScanID) to account for batch effects. Intra-sample correlation was modeled using duplicateCorrelation with the individual dog/specimen identities used as the blocking factor. Contrasts were specified for pairwise group comparisons, and empirical Bayes moderation was applied. *p*-values were adjusted using the Benjamini–Hochberg method. Differentially expressed genes (DEGs) and associated statistics (log2 fold changes, *p* values) were visualized with volcano plots (EnhancedVolcano, version 1.28.2). Hierarchical clustering and heatmap visualization of statistically significant DEGs (*p* < 0.05 or BH-adjusted FDR *p* < 0.05) were performed using the pheatmap (Pretty Heatmaps, version 1.0.13) R package.

Intersection analysis of DEG sets was conducted with the VennDetail (version 1.26.0) R package to identify, visualize (Venn diagrams), and extract expression details from unique and/or overlapping gene subsets. Gene Set Enrichment Analysis (GSEA) [78] of ranked DEG lists (e.g., gene symbols with moderated t-statistic values from limma) was performed using the GSEAPreranked tool with default parameters [weighted enrichment statistic, 1000 permutations, mean division (meandiv) metric for calculation of normalized enrichment score (NES)] and the Hallmark (H) and C6 (oncogenic signatures) MSigDB gene sets [79]. Enrichment results from selected analyses were compared and visualized with scatterplots (ggplot2, version 4.0.2) of the normalized enrichment scores for the gene sets in each analysis to visualize concordance and/or divergence in pathway-enrichment patterns. Mixed cell deconvolution of the TME was performed on Q3-normalized ROI expression data using the SpatialDecon R script (NanoString, Bruker Spatial Biology, Bothell, WA, USA) and the SafeTME cell profile matrix consisting of the expression profiles for 18 immune and stromal cell types d The outputs of the SpatialDecon algorithm include estimated abundance and proportions for each cell type within each ROI. ROC analysis was performed using the composite six-gene signature score and metastatic status with the pROC R package.

### 4.4. Quantitative Real Time PCR Validation

For each target gene, two primers and an internal hydrolysis fluorescent labeled probe (5′ end, reporter dye FAM (6-carboxyflourescein), 3′ end, quencher MGB (Minor Grove Binder, Applied Biosystems, Thermo Fisher Scientific, Waltham, MA, USA)) was ordered from Thermo Fisher Scientific (Thermo Fisher Scientific, Waltham, MA, USA). *PTEN* (Cf02628582_m1), *JAK1* (Cf02631294_m1), *LOC477699* (Cf02634791_m1), and *GAPDH* (Cf04419463_gH) were used.

One 20 µm section of FFPE was used for extraction following the QIAamp DNA FFPE Tissue Kit (Qiagen, Valencia, CA, USA). The standard operating protocol was followed with no modification. Briefly, 1 ml of xylene was added to each sample to dissolve the paraffin, 180 µl of ATL was added to each sample and was incubated at 56 °C for 1 h and then 90 °C for a second hour. After the incubations, 200 µl of AL and ethanol were added, and the sample was spun through on the column. The column was washed, and total nucleic acid was eluted in 60 µl of water.

The SuperScript^TM^ IV VILO^TM^ Master Mix with ezDNase^TM^ Enzyme (Invitrogen, Applied Biosystems, Thermo Fisher Scientific, Waltham, MA, USA) was used for cDNA synthesis following the manufacturer’s directions with the following modifications. Ten microliters of RNA were digested with 0.5 µl of 10X eZ DNase buffer and 0.5 µl of DNase enzyme by incubation at 37 °C for two minutes and then briefly centrifuged. Genomic DNA contamination was tested by using 1 µl of digested RNA and running the Real-time PCR housekeeping gene. Then, 2 µl of SuperScript IV VILO master mix and 6 µl water was added and incubated at 25 °C for 10 min, and then incubated at 50 °C for 10 min. The samples were inactivated at 95 °C for 3 min, chilled, and 80 µl of water was added.

Each PCR reaction contained 20x primer and probes for the respective qPCR system with a final concentration of 400 nM for each primer and 80 nM for the TaqMan probe and commercially available PCR mastermix (TaqMan Universal PCR Mastermix, Thermo Fisher Scientific) containing 10 mM Tris-HCl (pH 8.3), 50 mM KCl, 5 mM MgCl_2_, 2.5 mM deoxynucleotide triphosphates, 0.625 U AmpliTaq Gold DNA polymerase per reaction, 0.25 U AmpErase UNG (Applied Biosystems, Thermo Fisher Scientific, Waltham, MA, USA) per reaction and 5 µl of the cDNA sample in a final volume of 12 µl. The samples were placed in 384 well plates and amplified in an automated fluorometer (QuantStudio 7 Pro, Thermo Fisher Scientific). Thermo Fisher Scientific’s standard amplification conditions were used: 2 min at 50 °C, 10 min at 95 °C, 40 cycles of 15 s at 95 °C and 60 s at 60 °C. Fluorescent signals were collected during the annealing temperature and CT values extracted with a threshold of 0.1 and baseline values of 3–10.

Final quantitation was done using the comparative Cq method (User Bulletin #2, Applied Biosystems) and is reported as relative transcription or the n-fold difference relative to a calibrator. In brief, the reference gene was normalized to the Cq values of the target genes (ΔCq). The ΔCq was calibrated against the average of the negative control sample within each target gene. The linear amount of target molecules relative to the calibrator was calculated by 2^−ΔΔCt^. Therefore, all gene transcription is expressed as an n-fold difference relative to the calibrator. For comparison average of the target group (pN+) was compared to average of the control group (pN0) to mirror the comparisons made with the transcriptomic data.

### 4.5. Comparison of Canine Results to Human Samples

We compared our results to previously published data [33] on human cancer specimens collected and archived at the UC Davis Medical Center under IRB-approved protocol #1841486 that underwent Nanostring GeoMx^®^ Digital Spatial Profiling. Briefly, Primary tumor tissue and adjacent tumor microenvironment (TME) were excised from formalin-fixed paraffin-embedded (FFPE) blocks and re-embedded to generate tumor microarrays (TMAs). TMAs were sectioned for spatial transcriptomic analysis by the UC Davis Center for Genomic Pathology Laboratory (CGPL). Spatial transcriptomic profiling of both tumor and TME ROIs was performed in a single experimental workflow using the GeoMx^®^ Human Whole Transcriptome Atlas assay at the UC Davis Genomics Shared Resource (GSR). Morphology markers from the GeoMx^®^ Solid Tumor TME Morphology Kit, Pan-cytokeratin (PanCK), CD45, and SYTO 13, were applied. Fluorescent images were acquired at 20X magnification using the GeoMx^®^ Digital Spatial Profiler (DSP). Parallel fluorescent and H&E-stained serial sections guided region-of-interest (ROI) selection. PanCK-dominant Tumor ROIs were defined as previously reported [33], while TME ROIs were defined by areas of high CD45^+^ staining. Libraries were generated using GeoMx Seq Code Illumina-compatible primers, quality-checked, and sequenced on an Illumina NovaSeq X Plus system. FASTQ files were processed using the GeoMx^®^ NGS Pipeline Software. Following quality control, expression data were normalized using Q3 normalization. Tumor ROI data from this profiling run were previously published [33]. The TME ROI data presented here is newly analyzed and reported for the first time.

For further clarification, the canine data was also compared to publicly available mRNA expression and clinical outcome data retrieved from the Head and Neck Squamous Cell Carcinoma (HNSCC) Firehose Legacy dataset of The Cancer Genome Atlas (TCGA). Data were accessed through cBioPortal [80,81,82] and the Human Protein Atlas [69]. Optimal expression cutoffs for survival analyses were computed in R (v4.3.1) using maximally selected log-rank statistics. For each gene, all unique expression values were evaluated as candidate dichotomization thresholds using the log-rank test implemented in the survival package (v3.5-7). Thresholds producing groups of insufficient size were excluded. The optimal cutoff was defined as the threshold yielding the minimum log-rank *p*-value.

## 5. Conclusions

There is a distinct molecular signature in pN0 vs. pN+ canine HNSCC with downregulation of *PTEN*, *EST11*, *CD99*, *MAPKAPK2*, *OAZ1*, and upregulation of *LOC477699* representing potential biomarkers for risk stratification. Reduced JAK1 expression and endothelial cell abundance also appears to impact the propensity for locoregional spread and permissive change within the lymph node and may represent a therapeutic target for neoadjuvant/adjuvant intervention in dogs. Similar trends were observed in humans with downregulation of *PTEN*, *CD99*, *MAPKAPK2*, and *OAZ1* warranting further evaluation. Normal tissue next to metastatic tumors (pN+) has shifted molecular signatures from normal tissue next to more conservative lesions (pN0) lesions, potentially representing field cancerization. Future work will focus on prospective validation of these findings through use of a three-component risk score on the primary tumor.

## Figures and Tables

**Table 1:** Patient and tumor demographics for included samples.

	Dog Signalment	Tumor Size	Tumor Location	Locoregional Metastasis, Nature of Pathologic Staging Performed	Distant Metastasis, Staging Performed
1	16 yo MC Yorkshire Terrier	T2	Ventral tongue *	Macrometastasis bilateral MRLN, bilateral dissection of the MLN and MRLN	No, thoracic radiographs
2	12 yo MC Shih Tzu	T3	Left caudal mandible	Macrometastasis left MRLN, ipsilateral dissection of MLN and MRLN	No, thoracic radiographs
3	6 yo MC Pit Bull Terrier	T3	Left rostral to caudal mandible	Macrometastasis of left MLN with ENE, ipsilateral dissection of MLN and MRLN	No, thoracic CT
4	13 yo MC Maltese	T3	Left mandibular lip	Micrometastasis of left MLN, SLNB	No, thoracic CT
5	5 yo FS Alaskan Malamute	T3	Right caudal mandible	Macrometastasis right MLN and RMLN, necropsy	No, necropsy
6	8 yo MN Redtick Coonhound	T3	Right caudal mandible	Macrometastasis right MLN, necropsy	Yes, necropsy
7	13 yo FS Maltese	T3	Right Rostral-caudal mandible	None, bilateral dissection of the MLN and MRLN	No, thoracic CT
8	10 yo FS Chinese Crested	T2	Right caudal mandible	None, SLNB negative	No, thoracic CT
9	6 yo MC Siberian Husky	T1	Right Rostral Mandible	None, SLNB negative	No, thoracic CT
10	7 yo FS Labrador Retriever	T2	Right rostral mandible	No, SLNB negative	No, thoracic CT
11	12 yo MC Boxer Mix	T3	Bilateral rostral mandible	None, bilateral dissection of the MLN and MRLN	No, thoracic CT
12	10 yo MC Pit Bull	T3	Right caudal maxilla	None, ipsilateral dissection of MLN and MRLN	No, thoracic CT
13	7 yo MC Labrador Retriever	T1	Left caudal mandible	None, SLNB negative	No, thoracic CT
14	7 yo MI Irish Setter	T3	Caudal mandible	Macrometastasis L MLN, bilateral dissection of the MLN and MRLN	No, Thoracic CT
15	6 yo FS Husky Mix	T3	Ventral tongue	Macrometastasis bilateral MRLN, bilateral dissection of MLN and MRLN	
16	3 yo MC Golden Retriever	T1	Rostral maxilla	No, diagnostic imaging and cytology bilateral MLN, long-term follow up with no locoregional failure *	No, thoracic CT
17	2 yo MC Australian Shepherd	T3	Caudal mandible	No, diagnostic imaging and cytology bilateral MLN, long-term follow up with no locoregional failure *	No, thoracic CT
18	10 yo MC Pitbull	T2	Rosteal Maxilla	No, diagnostic imaging and cytology bilateral MLN, long-term follow up with no locoregional failure *	No, thoracic radiographs

Yo: year old; MC: male castrated; FS: female spaced; T1: <2 cm; T2: 2–4 cm; T3: >4 cm; SLNB: sentinel lymph node biopsy; MLN: mandibular lymph node; MRLN: medial retropharyngeal lymph node; ENE: extranodal extension; CT: computed tomography. * These samples were not included in the transcriptomic analysis.

## Data Availability

The original canine data presented in the study are openly available at the NCBI Gene Expression Omnibus (GEO) at https://www.ncbi.nlm.nih.gov/geo, GEO accession number: GSE346824.(accessed on 10 August 2026).

## Supplementary Materials

The following supporting information can be downloaded at: https://www.mdpi.com/article/10.3390/ijms27209163/s1.

## Abbreviations

The following abbreviations are used in this manuscript:

HNSCC	head and neck squamous cell carcinoma
pN0	pathologically normal lymph node
pN+	pathologically metastatic lymph node
TME	tumor microenvironment
DEGs	differentially expressed genes
FDR	false discovery rate
ROI	regions of interest
