# Supplementary material for: Spatial Molecular Signatures of Canine Oral Squamous Cell Carcinoma with and Without Locoregional Metastasis with Cross-Species Comparison to Human Samples
Source: Int J Mol Sci. 2026 Oct 15;27(20):9163. doi: 10.3390/ijms27209163 (PMC13705180; doi:10.3390/ijms27209163)
Supplement: Supplementary file 1 [file ijms-27-09163-s001.zip › ijms-4557645-supplementary/supplemental_/Supplementary 6A.pdf]

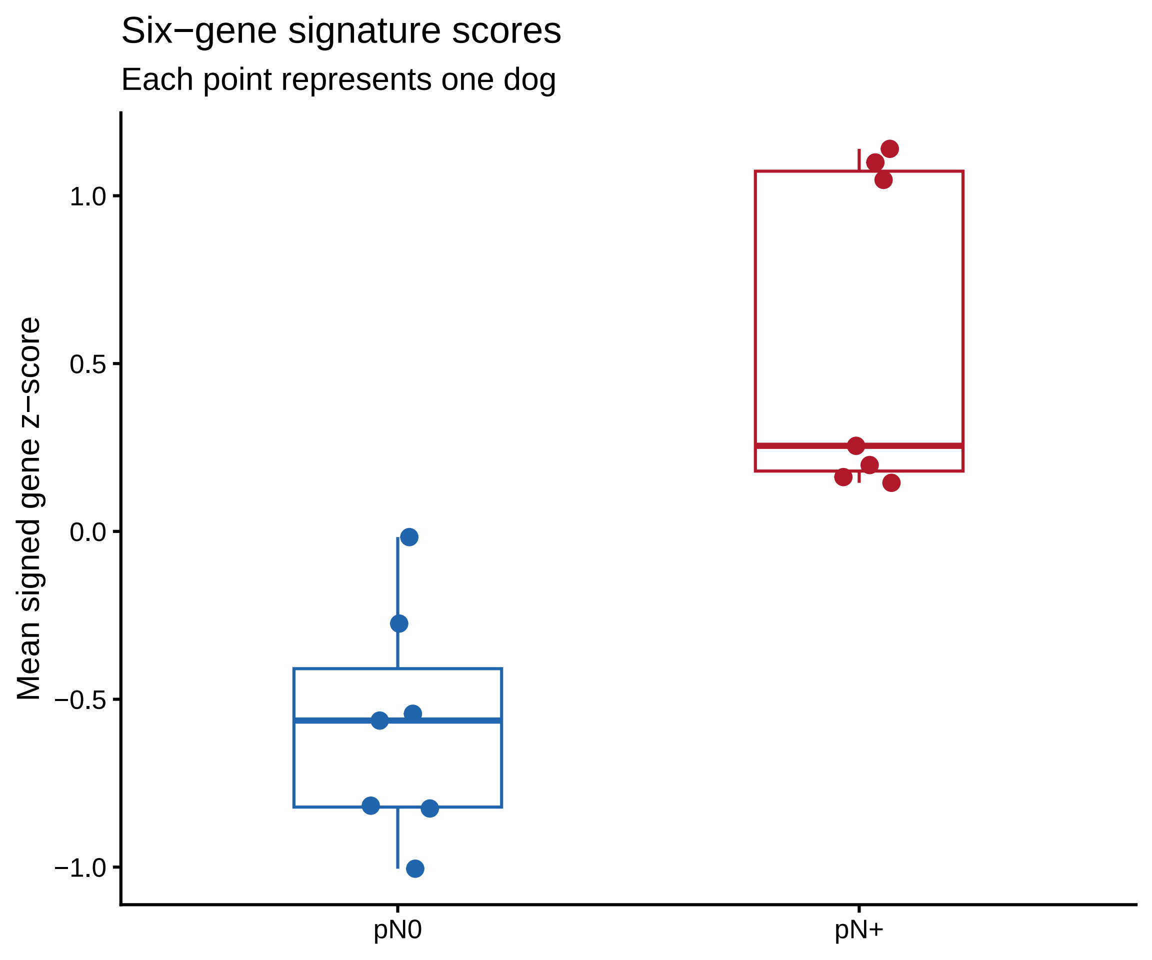
Six−gene signature scores
Each point represents one dog
Mean signed gene z−score
1.0
0.5
0.0
−0.5
−1.0
pN0
pN+
