# Supplementary material for: Spatial Molecular Signatures of Canine Oral Squamous Cell Carcinoma with and Without Locoregional Metastasis with Cross-Species Comparison to Human Samples
Source: Int J Mol Sci. 2026 Oct 15;27(20):9163. doi: 10.3390/ijms27209163 (PMC13705180; doi:10.3390/ijms27209163)
Supplement: Supplementary file 1 [file ijms-27-09163-s001.zip › ijms-4557645-supplementary/supplemental_/Supplementary6B.pdf]

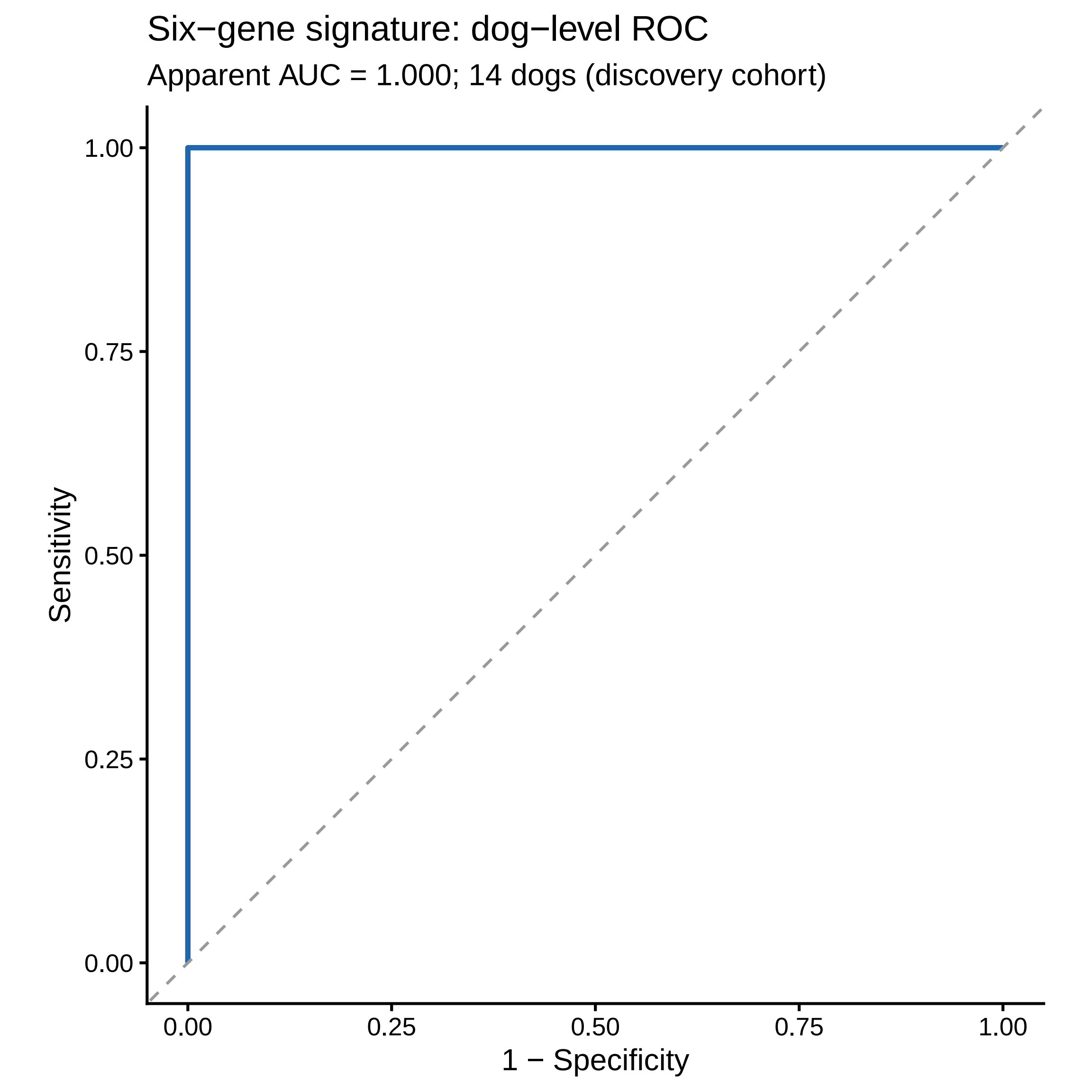

Six−gene signature: dog−level ROC
Apparent AUC = 1.000; 14 dogs (discovery cohort)
Sensitivity
1.00
0.75
0.50
0.25
0.00
0.00
0.25
0.50
0.75
1.00
1 − Specificity
