# Supplementary material for: Spatial Molecular Signatures of Canine Oral Squamous Cell Carcinoma with and Without Locoregional Metastasis with Cross-Species Comparison to Human Samples
Source: Int J Mol Sci. 2026 Oct 15;27(20):9163. doi: 10.3390/ijms27209163 (PMC13705180; doi:10.3390/ijms27209163)
Supplement: Supplementary file 1 [file ijms-27-09163-s001.zip › ijms-4557645-supplementary Table.pdf]

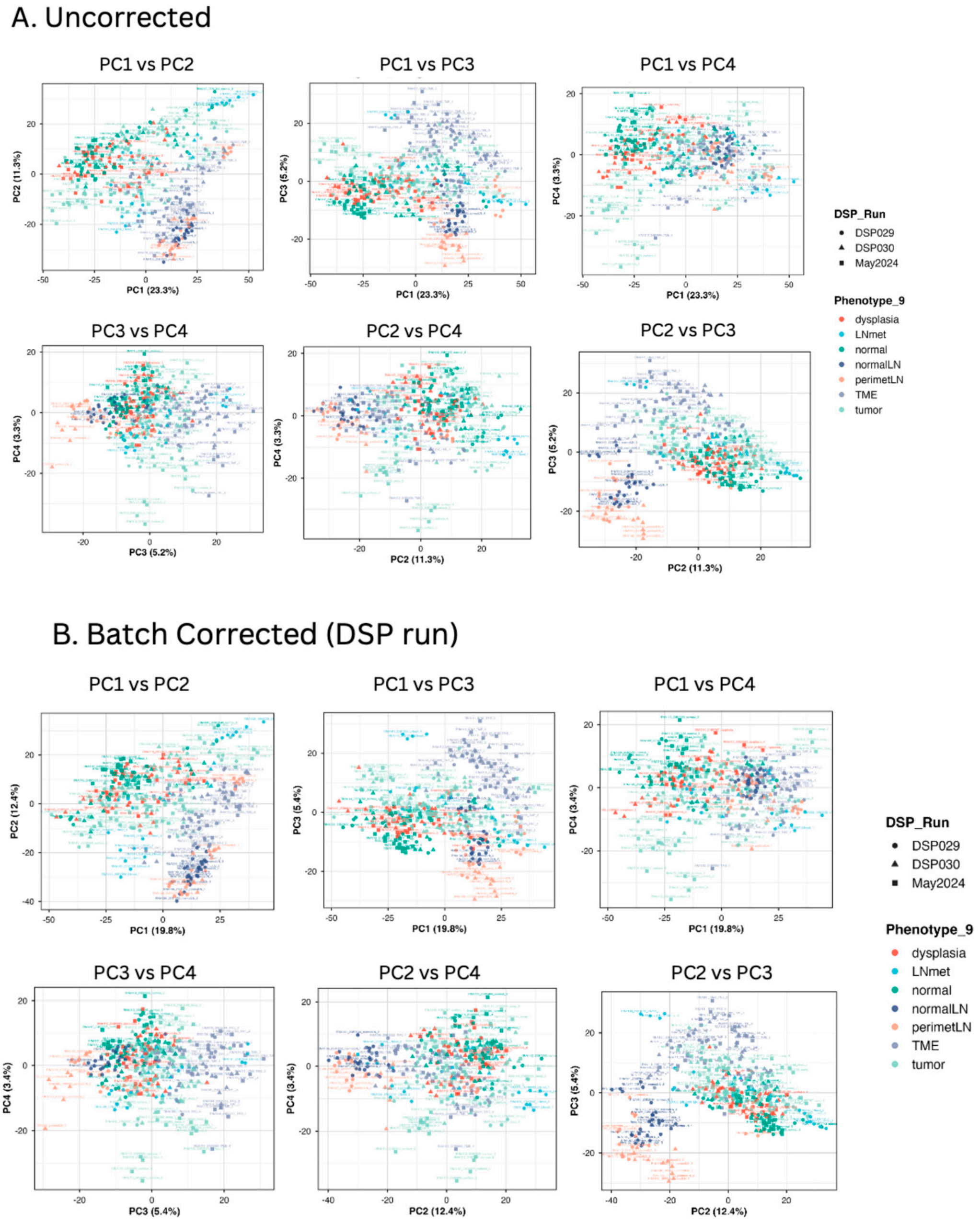

Supplementary Figure S1. PCA of all OSCC ROI expression data before and after batch correction. PCA was performed on expression data from all ROIs in the study *A)* before batch correction (uncorrected) and *B)* after batch correction for DSP Run. The results are shown as pairwise PCA plots of the first four principal components (PC1-PC4). DSP run is indicated by point shape, and the seven tissue/phenotypes are indicated by color, as shown in the figure legend.

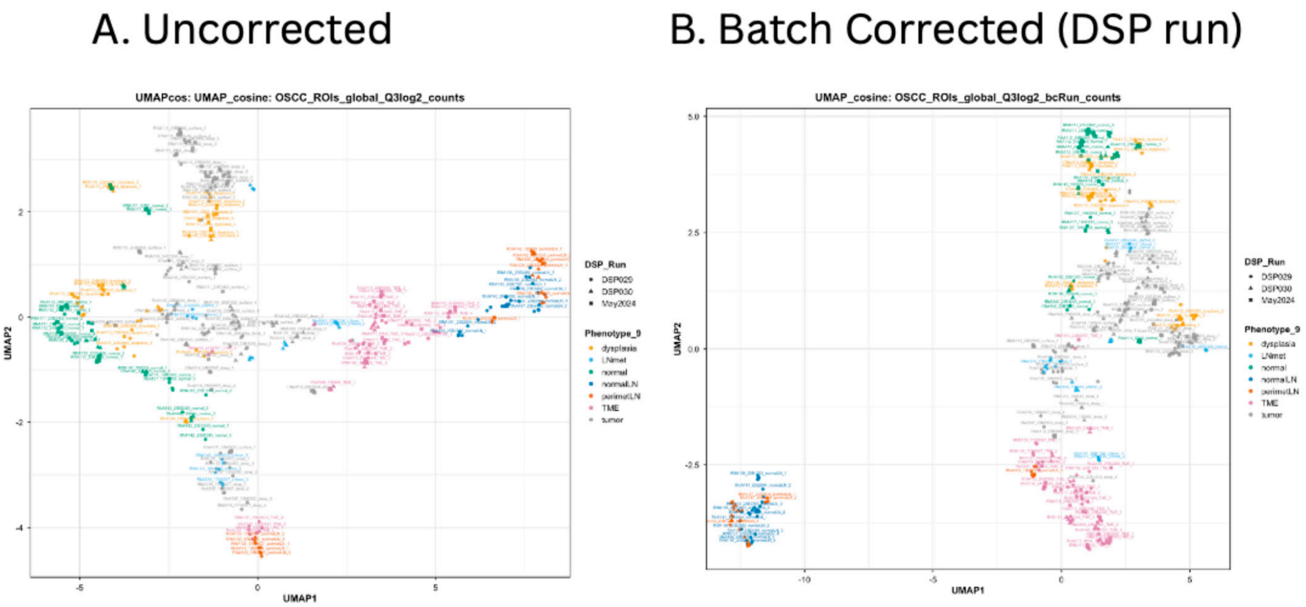

Supplementary Figure S2. UMAP analysis of all OSCC ROI expression data before and after batch correction. UMAP analysis was performed on expression data from all ROIs in the study *A)* before batch correction (uncorrected) and *B)* after batch correction for DSP Run. The results are shown as two-dimensional UMAP plots of the individual ROIs represented according to their UMAP1/2 coordinates. DSP run is indicated by point shape, and the seven tissue/phenotypes are indicated by color, as shown in the figure legend.

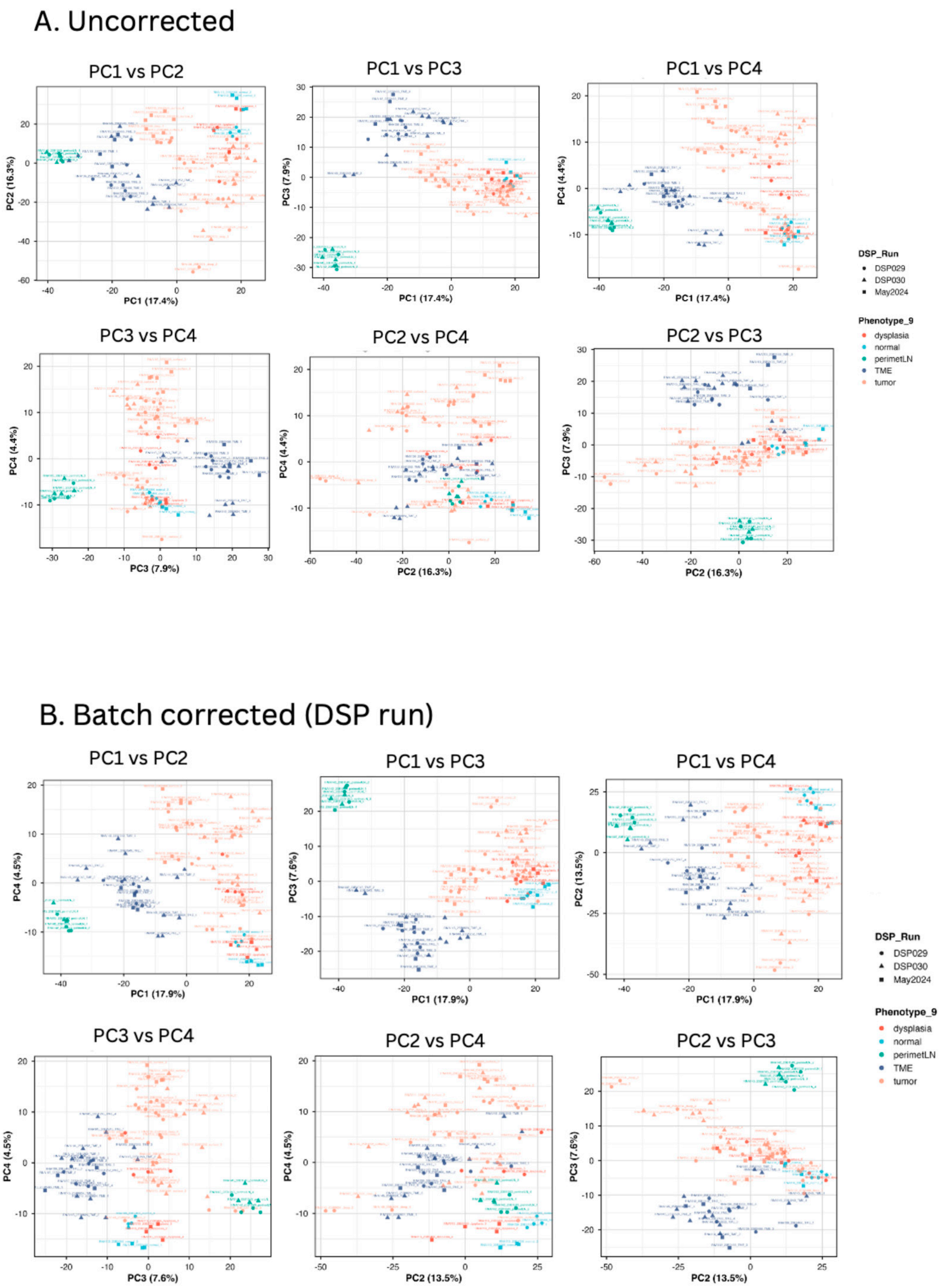

Supplementary Figure S3. PCA of anchor sample ROI expression data before and after batch correction. PCA was performed on expression data from the anchor sample ROIs in the study *A)* before batch correction (uncorrected) and *B)* after batch correction for DSP Run. The results are shown as pairwise PCA plots of the first four principal components (PC1-PC4). DSP run is indicated by point shape, and the seven tissue/phenotypes are indicated by color, as shown in the figure legend.

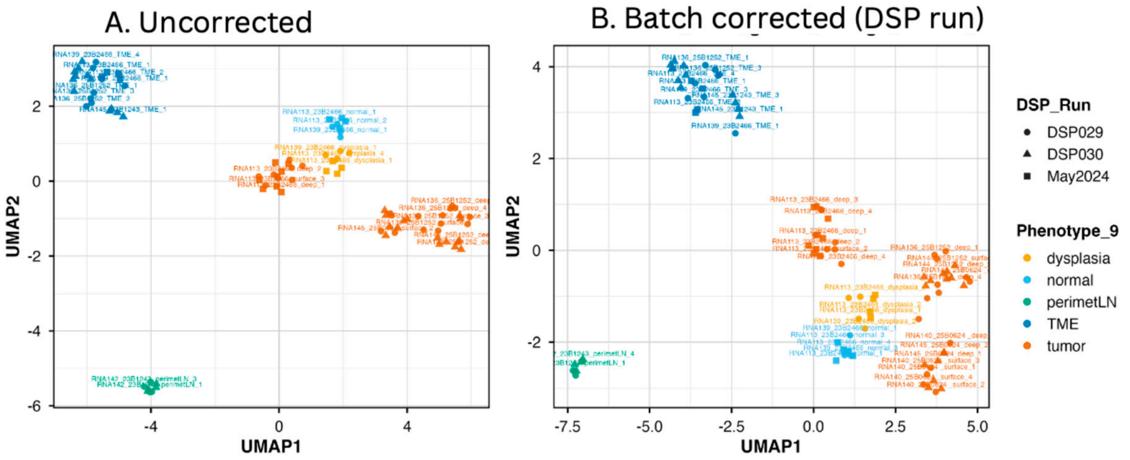

Supplementary Figure S4. UMAP analysis of anchor sample ROI expression data before and after batch correction. UMAP analysis was performed on expression data from the anchor sample ROIs in the study ***A)*** before batch correction (uncorrected) and ***B)*** after batch correction for DSP Run. The results are shown as two-dimensional UMAP plots of the individual ROIs represented according to their UMAP1/2 coordinates. DSP run is indicated by point shape, and the seven tissue/phenotypes are indicated by color, as shown in the figure legend.

A

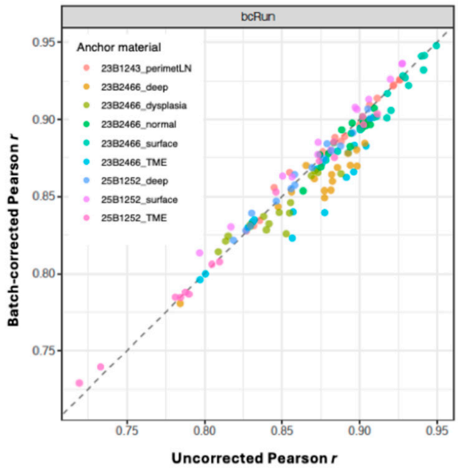

B

Median Pearson correlations of cross-run ROI pairs within each anchor sample

		-- Median Pearson Correlation --	
**Anchor Sample**	**Run Pair**	**Uncorrected**	**DSP Run**
23B1243_perimetLN	DSP029 vs DSP030	0.889	0.888
23B2466_TME	DSP029 vs May2024	0.865	0.840
23B2466_deep	DSP029 vs May2024	0.882	0.863
23B2466_dysplasia	DSP029 vs May2024	0.845	0.835
23B2466_normal	DSP029 vs May2024	0.895	0.894
23B2466_surface	DSP029 vs May2024	0.919	0.911
25B1252_TME	DSP029 vs DSP030	0.828	0.830
25B1252_deep	DSP029 vs DSP030	0.864	0.866
25B1252_surface	DSP029 vs DSP030	0.885	0.896

Supplementary Figure S5. Correlation analysis of anchor sample ROIs demonstrates high concordance of expression profiles before and after batch correction for DSP Run. A) Pearson correlation analysis was performed using anchor sample ROI expression data before (uncorrected) and after batch-correction for DSP Run. Cross-run comparisons were performed between ROIs derived from the same biological material (e.g., 23B2466_TME) to measure similarity of expression patterns across experimental runs. Correlation coefficients (Pearson *r*) were calculated and the results are depicted in the scatter plot to visualize the correlation coefficients obtained from the uncorrected and batch-corrected data for each ROI comparison. Each point represents one cross-run comparison The dashed diagonal represents the line of identity; points above or below the line indicate increased or decreased correlation, respectively, following batch correction. ***B)*** Median Pearson correlations of cross-run ROI pairs within each anchor sample. The median of the pairwise ROI correlations for each anchor sample and DSP run combination were determined and presented in the table above.

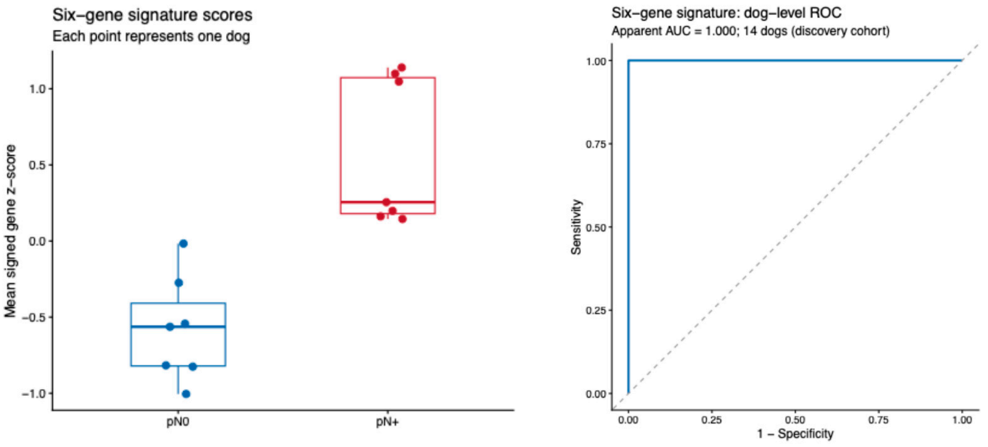

Supplementary Figure S6. Receiver operating characteristic (ROC) analysis of the 6-gene signature associated with pN+ status. Differential gene expression analysis (limma) of pN+ vs. pN0 tumors identified six differentially expressed genes (FDR p $\leq$ 0.05): *PTEN, EPSTI1, CD99, MAPKAPK2, OAZ1, LOC477699*. To assess the collective discriminatory power of this 6-gene signature for pN+ status, exploratory patient-level ROC analysis of the expression dataset was performed using the pROC R package. *A)* A composite 6-gene signature was calculated for each dog. Each point represents 1 dog, and boxplots depict the distribution of signature scores for dogs in each phenotypic group (i.e., pN0, pN+). *B)* ROC analysis was performed using the composite six-gene signature score and metastatic status with the pROC R package. The six-gene signature demonstrated complete separation of pN0 and pN+ dogs in this study's cohort and corresponded to an area under the ROC curve (AUC) of 1.000 (n = 14). Because the six genes were identified and evaluated using the same discovery cohort and that the cohort size was small, we consider this as an exploratory analysis, and independent validation will be required to determine the generalizability of the signature.

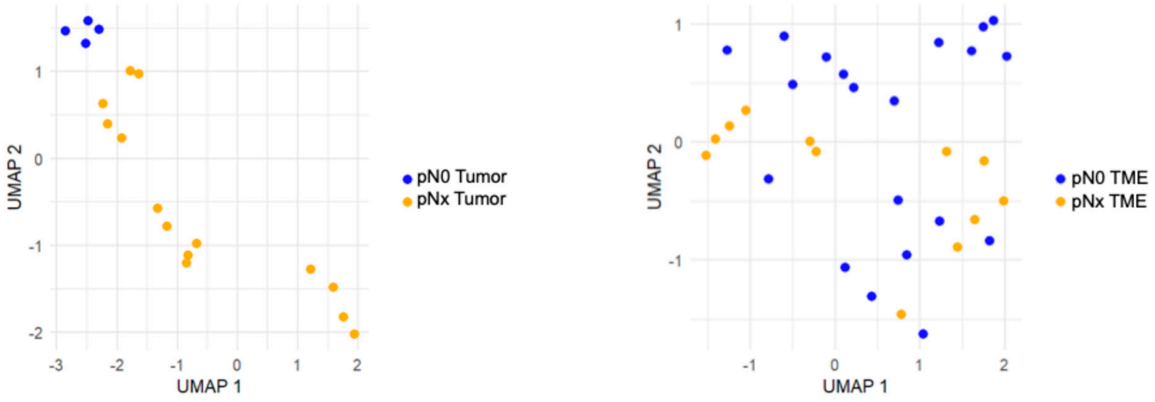

Supplemental Figure S7. UMAP of Tumor and TME ROI in the human GeoMx data set. Note the clear separation of the pN0 and pNx ROI (left) and the overlap of the TME ROI in the pN0 and pNX ROI.

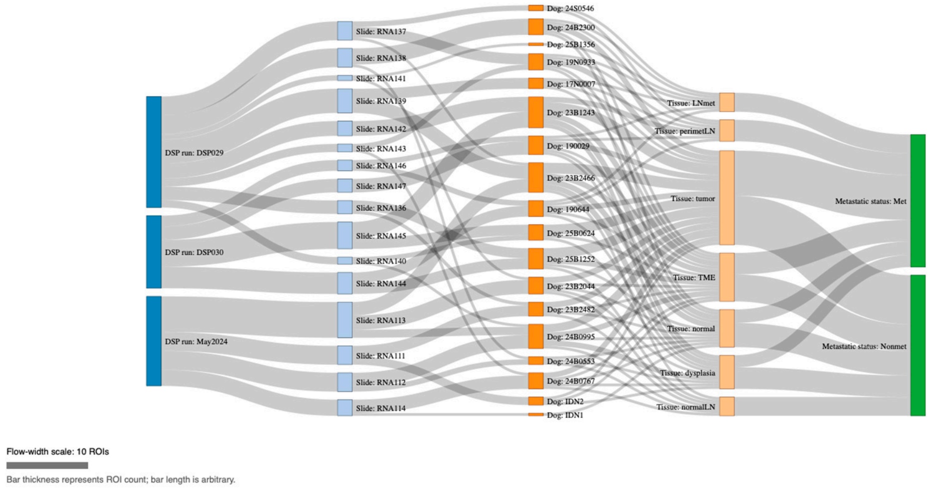

Supplementary Figure S8. Experimental design and flow of ROIs from DSP Run to Slide/Scan to Dog ID (Pathology Number) to Tissue type to Metastatic status. The flow-width scale indicates the ROI count in each flow.

Supplementary Table S1. Patient data for human samples included in comparative analysis.

Age	Sex	Ethnicity	Site	Stage	Perineural / Lymphovascular Invasion	Depth of Invasion	Grade	Extra Nodal Extension	Locoregional Disease	Distant Disease	Death	Smoking	Alcohol
62	F	Asian	Oral tongue	pT3N0	PNI-/LVI-	12mm	G2 (mod)	n/a	No	No	No	Current (15 pack years)	Rare
23	F	Asian	Oral tongue	pT3N0	PNI-/LVI-	8mm	G1 (well)	n/a	No	No	No	Never	None
30	M	White	Oral tongue	cT4aN1	PNI+/LVI-	Unk	G2 (mod)	Unk	Yes	Yes (at presentation)	Yes (6/13/21)	Never	None
73	M	White	Oral tongue	pT4aN3b	PNI+/LVI-	11.5mm	G2 (mod)	Yes (<2mm)	No	No	No	Former (30 years)	Occasional
34	M	White	Oral tongue	pT4aN3b	PNI+/LVI+	18mm	G1-3 (well-poor)	Yes (>2mm)	No	Yes (8/9/21)	Yes (8/18/21)	Never smoker (uses marijuana)	None
81	M	White	Maxilla	pT4aN2c	PNI-/LVI-	14mm	G2 (mod)	No	Yes (5/6/21)	No	Yes (3/15/23)	Former (30 years)	Yes (8-10

			alveolar										drinks/ week)
67	F	White	Mandible alveolar	pT4aN3b	PNI+/LVI-	22mm	G1 (well)	Yes (>2mm)	Yes (1/21/22)	No	Yes (3/21/22)	Former (56 years, quit 2 months prior)	Former
77	F	White	Retromolar trigone	pT4aN3b	PNI+/LVI+	15mm	G3 (poor)	Yes (>2mm)	No	No	No	Former (20 years)	None
68	F	White	Retromolar trigone	pT4aN2b	PNI+/LVI+	20mm	G2 (mod)	No	No	No	Yes (1/12/22)	Former (40 years)	Yes (1-2 drinks/ week)
30	F	White	Larynx	pT4aN0	PNI-/LVI-	Unk	G1 (well)	n/a	No	No	No	Never	None
28	M	White	Oral tongue	pT4aN0	PNI+/LVI-	20mm	G2 (mod)	n/a	No	No	No	Current (minimal; 4 pack years)	None
45	F	White	Buccal	pT2N0	PNI+/LVI-	7mm	G1 (well)	n/a	No	No	No	Never	Rare
33	M	White	Oral tongue	pT4aN3b	PNI+/LVI+	34mm	G2 (mod)	Yes (>2mm)	Yes (2/15/23)	Yes (2/15/23)	Yes (6/17/23)	Former (minimal; 5 years)	Rare
75	F	White	Oral tongue	pT2N0	PNI+/LVI-	7mm	G1 (well)	n/a	No	No	No	Former (minimal; 6 years)	Rare
65	M	White	Oral tongue	pT3N0	PNI+/LVI-	2mm	G2 (mod)	n/a	No	No	No	Current (45 pack years)	Yes (20 drinks/ week)

Supplementary Table S2 – Shared GSEA gene signatures on the C2, C6, and GO gene sets between pN+ tumors and TME between species.

Share pN+ Tumor pathways	NES (Human)	NES (Canine)	FDR q-val (Human)	FDR q-val (Canine)
CROMER_TUMORIGENESIS_UP	2.26	1.73	0.001	0.163
ANASTASSIOU_MULTICANCER_INVASIVENESS_SIGNATURE	2.04	1.92	0.007	0.047
REACTOME_COLLAGEN_DEGRADATION	2.04	1.53	0.007	0.381
NABA_MATRISOME_PRIMARY_METASTATIC_COLORECTAL_TUMOR	1.98	1.47	0.01	0.479
REACTOME_NCAM1_INTERACTIONS	1.97	1.68	0.011	0.212

NABA_CORE_MATRISOME	1.94	1.66	0.013	0.214
NABA_BASEMENT_MEMBRANES	1.93	1.56	0.014	0.328
TURASHVILI_BREAST_LOBULAR_CARCINOMA_VS_LOBULAR_NORMAL_DN	1.85	1.61	0.026	0.274
WESTON_VEGFA_TARGETS_6HR	1.67	1.7	0.072	0.195
NABA_ECM_GLYCOPROTEINS	1.61	1.67	0.102	0.222
WP_CHRONIC_HYPERGLYCEMIA_IMPAIRMENT_OF_NEURON_FUNCTION	1.58	1.52	0.113	0.409
WP_FOCAL_ADHESION_PI3KAKTMTORSIGNALING	1.58	1.61	0.114	0.279
REACTOME_NCAM_SIGNALING_FOR_NEURITE_OUT_GROWTH	1.53	1.54	0.135	0.365
VART_KSHV_INFECTION_ANGIOGENIC_MARKERS_DN	1.51	1.5	0.144	0.444
ROSS_AML_WITH_PML_RARA_FUSION	1.45	1.84	0.185	0.077
WP_MARKERS_OF_KIDNEY_CELL_LINEAGE	1.45	1.77	0.185	0.134
WESTON_VEGFA_TARGETS	1.45	1.77	0.185	0.13
WP_PI3KAKT_SIGNALING	1.38	1.48	0.231	0.479
KEGG_CYTOKINE_CYTOKINE_RECEPTOR_INTERACTION	1.35	1.7	0.249	0.198
ONDER_CDH1_TARGETS_2_UP	1.33	1.65	0.266	0.239
MARTENS_TRETINOIN_RESPONSE_UP	1.19	1.44	0.377	0.543
BMI1_DN.V1_UP	1.5	1.49	0.12	0.372
KRAS.600_UP.V1_UP	1.4	1.44	0.243	0.402
GOMF_EXTRACELLULAR_MATRIX_STRUCTURAL_CONSTITUENT	2.03	1.5	0.053	0.477
GOBP_POSITIVE_REGULATION_OF_EXTRINSIC_APOPTOTIC_SIGNALING_PATHWAY	1.97	1.45	0.057	0.541
GOBP_COLLAGEN_METABOLIC_PROCESS	1.92	1.45	0.069	0.539
GOBP_COLLAGEN_FIBRIL_ORGANIZATION	1.87	1.6	0.096	0.392
GOCC_EXTERNAL_ENCAPSULATING_STRUCTURE	1.82	1.43	0.111	0.563
GOCC_ENDOPLASMIC_RETICULUM_LUMEN	1.66	1.39	0.158	0.653
GOMF_SIGNALING_RECEPTOR_REGULATOR_ACTIVITY	1.64	2.03	0.166	0.041
GOBP_REGULATION_OF_RELEASE_OF_SEQUESTERED_CALCIUM_ION_INTO_CYTOSOL	1.53	1.59	0.234	0.41
GOBP_EPITHELIAL_CELL_DIFFERENTIATION_INVOLVED_IN_KIDNEY_DEVELOPMENT	1.49	1.47	0.249	0.5
GOBP_RESPONSE_TO_ACTIVITY	1.49	1.51	0.249	0.452
GOBP_NEURON_FATE_COMMITMENT	1.47	1.65	0.257	0.355
GOCC_PLATELET_ALPHA_GRANULE	1.46	1.48	0.26	0.492
GOCC_CELL_BODY	1.44	1.45	0.272	0.551
GOBP_CALCIUM_ION_TRANSMEMBRANE_IMPORT_INTO_CYTOSOL	1.43	1.44	0.273	0.56
GOBP_MORPHOGENESIS_OF_A_BRANCHING_STRUCTURE	1.39	1.36	0.293	0.693
GOBP_BLOOD_VESSEL_MORPHOGENESIS	1.38	1.36	0.301	0.696
GOBP_REPRODUCTIVE_SYSTEM_DEVELOPMENT	1.37	1.44	0.303	0.567
GOMF_CYTOKINE_RECEPTOR_BINDING	1.32	1.55	0.332	0.416
GOBP_SEX_DIFFERENTIATION	1.28	1.57	0.355	0.425

GOMF_SULFUR_COMPOUND_BINDING	1.27	1.48	0.365	0.495
**Shared PN+ TME**	**NES (Human)**	**NES (Canine)**	**FDR q-val (Human)**	**FDR q-val (Canine)**
NABA_MATRISOME_ASSOCIATED	1.880	1.790	0.0100	0.1240
ESC_V6.5_UP_LATE.V1_UP	1.950	1.500	0.0020	0.3530
KRAS.600_UP.V1_UP	1.410	1.450	0.0870	0.3010
GOBP_POSITIVE_REGULATION_OF_DEVELOPMENTAL_GROWTH	1.360	1.510	0.1820	0.7090
GOBP_REGULATION_OF_ENDOCRINE_PROCESS	1.580	1.530	0.0740	0.6930
GOBP_SPINAL_CORD_DEVELOPMENT	1.500	1.590	0.1080	0.6060
GOMF_GROWTH_FACTOR_ACTIVITY	1.440	1.860	0.1310	0.1510
GOMF_HORMONE_ACTIVITY	1.440	1.510	0.1330	0.7460

Supplementary Table S3: ROI per tissue compartment per patient included in analysis

ID	Metastatic Status	ROI Lymph Node Metastasis	ROI TME	ROI Perimet normal Lymp Node	ROI tumor	ROI dysplasia	ROI Normal epithelium	ROI Normal Lymph Node	Total_ ROIs	Slide ID
**17N0007**	Metastatic	4	4	4	4	0	0	0	**16**	RNA139
**190029**	Metastatic	4	4	4	8	4	4	0	**28**	RNA146, RNA147
**190644**	Metastatic	4	4	4	8	4	0	0	**24**	RNA144, RNA146
**19N0933**	Metastatic	4	4	4	8	0	4	0	**24**	RNA137, RNA143
**23B1243**	Metastatic	0	8	8	16	6	8	0	**46**	RNA142, RNA145
**23B2044**	Nonmetastatic	0	4	0	9	4	4	4	**25**	RNA113, RNA141
**23B2466**	Nonmetastatic	0	8	0	16	8	8	4	**44**	RNA113, RNA137, RNA139
**23B2482**	Nonmetastatic	0	4	0	9	0	4	4	**21**	RNA111, RNA136
**24B0553**	Nonmetastatic	0	0	0	0	4	4	4	**12**	RNA112, RNA138
**24B0767**	Nonmetastatic	0	4	0	8	4	4	4	**24**	RNA114, RNA143
**24B0995**	Nonmetastatic	0	4	0	10	4	4	4	**26**	RNA112, RNA113, RNA140
**23B1898**	Nonmetastatic				6	4			**10**	
**24B2300**	Metastatic	4	4	4	8	0	4	0	**24**	RNA138
**24S0546**	Metastatic	4	0	4	0	0	0	0	**8**	RNA137

**25B0624**	Metastatic	0	4	0	15	4	0	0	**23**	RNA140, RNA145
**25B1252**	Nonmetastatic	0	8	0	15	0	4	4	**31**	RNA136, RNA144
**25B1356**	Metastatic	4	0	0	0	0	0	0	**4**	RNA141
**IDN1**	Nonmetastatic	0	0	0	0	0	4	0	**4**	RNA114
**IDN2**	Nonmetastatic	0	4	0	4	4	0	0	**12**	RNA111
**Total number of ROIs (post QC filtering, removal of ROIs with questionable phenotype)**									**406**	

Supplementary Table S4. Read counts through GeoMX NGS pipeline for the new canine dataset (Run 2-3, not previously published)

Description	Raw Reads	Trimmed Reads	Aligned Reads	Deduplicated Reads	Stitched Reads
**Totals per experiment:**	2,205,594,421	2,194,699,448	2,104,514,986	57,534,841	2,178,247,593
**Mean:**	4,978,768.44	4,954,174.83	4,750,598.16	129,875.49	4,917,037.46
**Median:**	3,961,716.00	3,945,610.00	3,794,955.00	99,322.00	3,924,281.00
**S.D.:**	3,882,545.94	3,868,350.52	3,702,695.62	104,144.26	3,835,261.46
